# Naming and Shaming for Conservation: Evidence from the Brazilian Amazon

**DOI:** 10.1371/journal.pone.0136402

**Published:** 2015-09-23

**Authors:** Elías Cisneros, Sophie Lian Zhou, Jan Börner

**Affiliations:** 1 Zentrum für Entwicklungsforschung, University of Bonn, Bonn, Germany; 2 Institute for Food and Resource Economics, University of Bonn, Bonn, Germany; 3 Zentrum für Entwicklungsforschung, University of Bonn, and Center for International Forestry Research (CIFOR), Bonn, Germany; National University of Singapore, SINGAPORE

## Abstract

Deforestation in the Brazilian Amazon has dropped substantially after a peak of over 27 thousand square kilometers in 2004. Starting in 2008, the Brazilian Ministry of the Environment has regularly published blacklists of critical districts with high annual forest loss. Farms in blacklisted districts face additional administrative hurdles to obtain authorization for clearing forests. In this paper we add to the existing literature on evaluating the Brazilian anti-deforestation policies by specifically quantifying the impact of blacklisting on deforestation. We first use spatial matching techniques using a set of covariates that includes official blacklisting criteria to identify control districts. We then explore the effect of blacklisting on change in deforestation in double difference regressions with panel data covering the period from 2002 to 2012. Multiple robustness checks are conducted including an analysis of potential causal mechanisms behind the success of the blacklist. We find that the blacklist has considerably reduced deforestation in the affected districts even after controlling for the potential mechanism effects of field-based enforcement, environmental registration campaigns, and rural credit.

## Introduction

Brazil stands out as one of the few countries in the world where tropical deforestation rates have dropped over the past decade [1]. Emerging evidence from quasi-experimental evaluation studies on the effectiveness of Brazil’s post-2004 strategy to combat Amazon deforestation unambiguously suggests that environmental policy has come to play a major role in determining land use decisions in the region [2–6]. In 2004, the Brazilian government has launched a Plan to Combat Deforestation in the Amazon (PPCDAM in its Portuguese acronym). The first two PPCDAM operated from 2004–8 and 2009–11, respectively, and the third PPCDAM ends in 2015. Clearly, the drop in Amazon deforestation from over 27 thousand sqkm in 2004 to less than 10 thousand sqkm since 2009 results from a myriad of factors including the effects of the 2008 global financial crisis on international commodity demand [7]. Arima et al. [5] provide a detailed account of the Brazilian environmental policy context including statistical analysis of the overall effect of the most recent policy measures on deforestation in the Amazon region. Here we build on the approach chosen by Arima et al. to study whether and how the list of priority municipalities (henceforth “district blacklist”) issued by the Brazilian Ministry of Environment since 2008 played a measurable role in reducing Amazon forest loss. Brazil has pioneered the use of blacklisting as a forest conservation policy strategy and understanding its effect can help us to assess the potential of transparency and accountability initiatives in the conservation sector. We find that, on average, blacklisted districts have experienced distinctly larger reductions in deforestation than comparable non-listed districts and produce evidence that this difference is partially a genuine effect of blacklisting.

The paper is structured as follows. First, we provide a brief background of the Brazilian forest policy context and describe key elements of the Brazilian blacklisting strategy. We also discuss the potential mechanisms and pathways through which blacklisting might have contributed to reducing deforestation beyond the combined effect of other policy instruments. Next we summarize our empirical strategy to estimate the effect of blacklisting on deforestation, highlighting the key differences between our approach and strategy used in [5]. After documenting our data sources we present main results and robustness checks. Subsequently, we discuss potential caveats of our analysis in the context of the emerging literature evaluating conservation programs and provide conclusions and implications for conservation policy design.

### Forest policy background

Apart from a substantial expansion of the region’s protected area network [6], field-based law enforcement operations targeted at deforestation hot-spots by using remote sensing technologies have shown to be important short-term success factors to forest conservation [3]. One of the reasons for the increased effectiveness of field-based enforcement has been an intense collaboration between the Brazilian Environmental Protection Agency (IBAMA) and the state-level Public Prosecutors (MP’s in its Portuguese acronym). MP’s have been shown to exert positive effects on environmental policy outcomes by enhancing legal coercion [8]. Especially in the state of Pará, the MP’s have been involved in enforcing property embargoes issued by IBAMA, where for example the MP’s have engaged with meat packers and supermarket chains that were previously purchasing beef from illegal sources [5].

In addition, several Amazon states pioneered by the State of Mato Grosso, introduced so called Rural Environmental Registries (CAR in its Portuguese Acronym) that were recently combined in a federal registration system. Through the CAR, landholders with and without formal property rights declare the size and spatial boundaries of their land holdings, which enhances the government’s ability to monitor compliance with the Brazilian Forest Code [9].

Complementary to government actions, measures to contain the effect of cropland expansion were also taken by the private sector [10]. The so called “Soy Moratorium” was an agreement among major soy bean traders to not buy soy grown on land that was cleared after July 2006. Evaluations of the Soy Moratorium have produced mixed results, with indirect land use change potentially compromising its effectiveness [5, 11].

Between late 2007 and early 2008, Brazil introduced additional measures to reinforce field-based enforcement action. First, resolution 3.545 published in 2008 by the Brazilian Monetary Council (Conselho Monetário Nacional) limits credit access to farms that are non-compliant with the Brazilian Forest Code and conditions future credit access on proofs of compliance with environmental legislation. Assunção et al. [12] estimate that this measure has avoided 2700 square kilometers of deforestation between 2009 and 2011. Second, the Presidential Decree 6.321 (December 2007) created the legal basis for the blacklist that contains districts with outstanding historical deforestation rates. In blacklisted districts, stricter rules with regard to the authorization of forest clearing applied and defined administrative targets had to be fulfilled in order to qualify for a removal from the list.

Both decrees essentially operate as cross-compliance measures by making access to credit (resolution 3,555) or authorization of forest clearings (Decree 6.321) conditional on compliance with forest law and registration requirements respectively. A major difference between the decrees is that the credit restriction applies to the whole Amazon biome, whereas only a subset of Amazon districts is blacklisted. This difference allows us to adopt the empirical strategy outlined in section 2.

#### History and impact logic of the Brazilian district blacklist

Decree 6.321, published in December 2007, clearly defines the objective of the blacklist as a strategy to monitor and control illegal deforestation and prevent land degradation. It states that the list is to be updated annually based on official deforestation statistics and specifies the complementary roles of IBAMA and the National Institute for Agrarian Reform (INCRA) in monitoring and registering landholdings in the blacklisted districts. Three criteria are put forward as being used to compose the blacklist, namely:

1. The total deforested area
2. The total deforested area in the preceding three years
3. The increase of deforestation of minimum three out of the past five years

**Fig 1:**
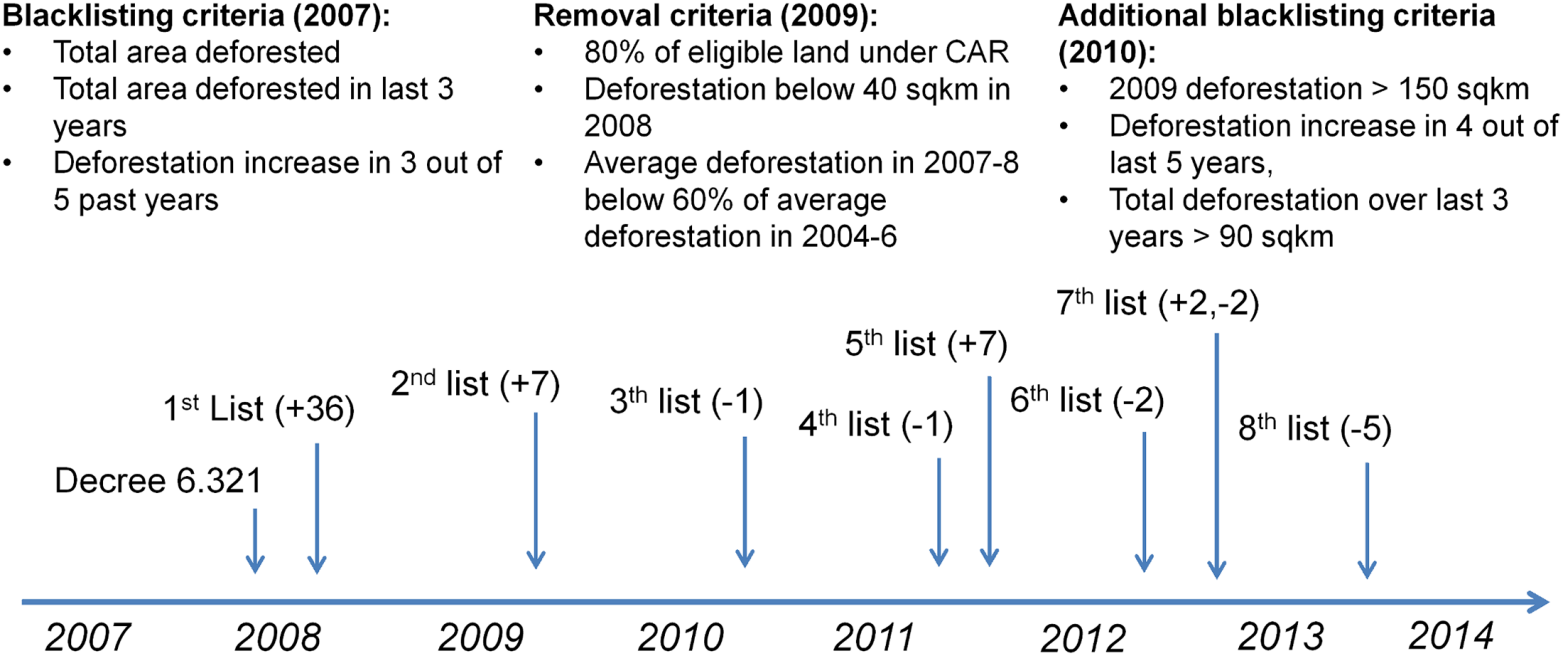
History of district blacklisting and blacklist criteria. Positive numbers in parentheses depict additions to the blacklist. Negative numbers depict removals.

Fig 1 schematically depicts how the blacklist has evolved since the publication of Decree 6.321.

In January 2008, the first blacklist was published covering 36 districts. Seven districts were added in both 2009 and 2011. The criteria for the removal from the blacklist were introduced in 2009. Removal was conditioned on registering at least 80% of the eligible area (mostly privately claimed land) under the CAR. Moreover, annual deforestation had to be kept below 40 sqkm. Only six districts were removed as of 2012.

District blacklisting probably qualifies as the most innovative element in Brazil’s multi-instrument conservation policy mix. To our knowledge no other country has yet applied a similar institutional cross-compliance mechanism in the forestry sector. The impact pathway of blacklisting is still unclear and very little research on blacklisting as a governance mechanism exists. Jacobs and Anechiarico [13] argue that contractor blacklisting is a sensible and ethically justifiable strategy to protect government organizations from fraud. China has experimented with an environmental disclosure policy including publication of lists of environmental regulation violators. A recent study found that this blacklisting strategy has helped in engaging civil society stakeholders in environmental governance [14]. The study, however, concluded that effects on behavioral change have been limited due to the country’s authoritarian structure. In 2010, a synthesis report by the Transparency and Accountability Initiative lists several largely untested assumptions regarding the impact channels of public disclosure policies. These include greater accountability through transparency and stimulation of action among a wide range of stakeholders. The report found that public disclosure policies have considerable potential to improve governance in sectors such as public service delivery, natural resource governance and donor aid [15]. Similar findings on public disclosure policies are reported by Blackman [16] and Tietenberg [17]. Among the potential impact channels of public disclosure policies, building internal and external pressure in favor of desired action was likely to be the most important motivation behind the Brazilian blacklist.

A multi-institutional evaluation of the Brazilian government’s Plan to Combat Deforestation in the Amazon (PPCDAM) concluded that “the priority list [blacklist]… turned out to be a cost-effective means to stimulate co-responsibility of district-level political elites—deforestation was ultimately also a problem of mayors and the local society” ([2], p. 41). Three distinct types of mechanisms have been recognized playing an important role:

1. **Administrative disincentive**: economic burden of administrative compliance measures motivate local stakeholders to take action. Specifically, landholdings in the blacklisted districts are required to obtain a georeferenced certification with INCRA as a precondition for authorized forest clearings.
2. **Reputational risk**: Public disclosure motivates action by public and private stakeholders as well as the civil society at district-level to “defend” the reputation of the district. The driving force behind this motivation could, for example, be concern about losing future business opportunities or increased environmental monitoring and enforcement action.
3. **External support/pressure**: Blacklisted districts may crowd in financial and logistic support from international NGO and public administrations creating incentives for improvements in local governance. On the other hand, blacklisted districts may also have attracted additional attention by national and subnational law enforcement agencies, such as the Brazilian Environmental Protection Agency (IBAMA).

We hypothesize that the administrative disincentive itself has played a minor role in promoting forest conservation. The blacklist introduces additional cost to farmers that depend on legal clearing permissions due to the obligation to register with INCRA. In consequence legal clearing rates may reduce. In districts where a considerable share of the forest and agriculture-based economy relies on legal forest use and conversion, the moratorium on new licenses for legal clearings may thus have increased registrations and reduced deforestation rates. On the other hand, if most of the land users in a district rely predominantly on illegal deforestation, additional conditions attached to obtaining environmental licenses will, all else equal, only have small effects on land users’ behavior.

Regarding the conditions to be removed from the blacklist, conditions such as the 80% CAR registration target are considered to be separate from the administrative disincentive channel. The reason for this is that these conditions do not exercise direct restrictions on the political administration nor on individual land users. Nonetheless, these rules generate costs if local stakeholders take action towards getting off the list. Such actions could be induced through both the reputational risk and through external support/pressure channels.

There is anecdotal evidence that reputational risk has played a significant role in bringing down deforestation in some of the blacklisted districts. In the district Paragominas (state of Pará), for example, a local stakeholder initiative to expand CAR registration and reduce deforestation rates was formed when the district appeared on the 2008 blacklist [18]. One of the motivations for the leaders of this initiative was reportedly the objective of “… reverting the negative image bestowed by being on the red-list [meaning blacklist]” ([18], pg. 23). Inspired by the “success” of Paragominas, the state of Pará launched a public support program (the Green Districts Program) for districts that achieved removal from the blacklist. Similar factors played a role in the district Brasil Novo that was removed from the list in 2013 (Environmental Secretary of Brazil Novo—public event, Brasilia, 7.10.2013).

The third group of mechanisms—external support/pressure—could also have played an important role. Both national and international NGOs have concentrated efforts to support CAR registration in blacklisted districts in collaboration with local and state-level government agencies. Support included both technical-scientific and local logistic measures to enable CAR registration at higher rates (The Nature Conservancy—interview, Belém, 4.10.2013). CAR registration exposes landholders to greater scrutiny by authorities and supporting NGO’s and thus also a higher risk of being held responsible for illegal deforestation. A recent study on the effect of CAR on deforestation has nonetheless produced ambiguous results with respect to deforestation outcomes [19]. In addition, blacklisted districts have been subject to more intense enforcement activities by IBAMA during (but also before) the blacklist was published. Moreover, blacklisting could have influenced rural credit flows into blacklisted districts as suggested by article 11 of Decree 6.321.

The empirical strategy described below is designed to measure the overall effect of the blacklisting policy on deforestation and to capture the causal effect of the third (and to some extent the second) group of mechanisms. The Brazilian blacklisting policy may have had a bearing on all three mechanism categories. But, the reputational risk and external support/pressure channels most closely represent the impact theory of public disclosure policies as discussed above [17].

## Empirical Strategy

The methodological challenge of evaluating the effect of the blacklist on deforestation in the blacklisted districts consists of identifying a counterfactual scenario of what would have happened in the absence of the blacklist [20]. From the previous section, we know that blacklisting was not random. Instead, regulators have used defined selection criteria that were linked to historical deforestation. Regression Discontinuity Design (RDD) is a commonly used evaluation technique for interventions where the selection mechanism is known [21] however unfortunately, only the three official criteria were made public, but not the exact approach, e.g. weighting used to arrive at the published blacklists. Although past deforestation highly correlates with selection, it is not possible to reproduce the first list of 36 districts based on the three published selection criteria alone. Considering the first blacklist criterion (see also S1 Fig), the 36 districts with the highest total forest loss as of 2007 include only 20 of the blacklisted districts. The 36 largest deforesters during the years 2005 to 2007 (second criterion) comprise only 25 of the blacklisted districts. Finally, 206 districts fulfill the criterion of three years of increments in deforestation during the past five years (2003–2007) and only 14 of them are blacklisted. Three blacklisted districts (Ulianópolis Paranaíta, Porto dos Gaúchos) did not fulfill any of the three criteria. Thus, we can only speculate which other criteria could have played a role in composing the blacklist. Moreover, our sample of treated districts is too small for informative local linear regression analyses in a RDD.

Our approach relies on panel data with annual observations of deforestation and other covariates over eleven years that complement the relatively low number of treated observations, i.e., only 50 districts were blacklisted as of 2012. Since most of our data is available only at district level, we choose the district as our unit of analysis. We first use a first difference (FD) estimation model to eliminate unobserved time-invariant district-level effects on deforestation. Second, we use matching on pre-blacklist characteristics to reduce model-dependence and the selection bias resulting from targeting the blacklist to districts with high deforestation rates [22]. We abstain from estimating treatment effects directly from the matched data set, because we expect a limited degree of common support. Instead we re-estimate our FD model using the matched data set. Third, we follow the strategy documented in Ferraro and Hanauer [23] to estimate the net treatment effect of blacklisting, i.e., the treatment effect after controlling for potential changes in external pressure and support measures (see previous section). This approach essentially blocks the effect that blacklist-induced changes in field-based enforcement intensity, rates of CAR registration, and rural credit flows could have had on deforestation in blacklisted districts. The remaining effect is called the net average treatment effect and captures all impact mechanisms other than the three above-mentioned causal mechanisms.

Following Jalan and Ravallion [24], we derive the double difference estimation model for our purpose as follows. Using yearly log deforestation (ln*D*_*it*_) as the outcome variable, the panel fixed effect can be written as:

$$\text{ln}D_{it}=\beta B_{it}+X\prime_{it}\gamma +tZ\prime_{i}\delta +\eta_{t}+\alpha_{i}+t\kappa_{s}+u_{it}$$ (1)

where *B*_*it*_ is the treatment variable indicating whether district *i* has been blacklisted at any time *t*, *X*_*it*_ is the vector for time-varying covariates, and *Z*_*i*_ is a vector of time-invariant covariates or the so-called “initial conditions”. Initial conditions are interacted with the time variable *t* and thus remain in Eq 2 below even after taking first differences. The underlying rationale is that the deforestation trend is likely to be affected by pre-treatment local conditions [24]. *α*_*i*_ is the district-specific fixed effect, which captures all time-invariant locally idiosyncratic influences on deforestation, year-specific effects *η*_*t*_ control for yearly changes to the deforestation trend, common to all districts, notably macroeconomic or environmental shocks and changes in the Brazilian environmental policies. State-specific effects *κ*_*s*_ capture differences in the implementation of federal laws on the state level. *u*_*it*_ denotes the error term. Both fixed effect and first difference estimators can be used, but we proceed with the first difference estimator that is less prone to serial correlation ([25], pg. 349). Taking first differences, Eq 1 becomes:

$$\Delta \text{ln}D_{it}=\beta \Delta B_{it}+\Delta X\prime_{it}\gamma +Z\prime_{i}\delta +\Delta \eta_{t}+\kappa_{s}+\Delta u_{it}$$ (2)

here the district-specific fixed effect is canceled out and the initial conditions stay in the equation as time-invariant covariates (Δ*t* = 1). In the first difference form, the year specific effects to the trend are transformed to standard year dummies Δ*η*_*t*_.

The treatment coefficient *β* measures the average treatment effect, i.e., the average change in deforestation due to blacklisting for all years after treatment (shift in deforestation trend). Deforestation is measured over the period from August to July (see S1 Text). Treatment indicators have to account for the fact that blacklists were released at different points in the year. The first list of 36 districts was published at the end of January 2008 (see Fig 1). Hence, we set treatment *B*_*it*_ to 0.5 to represent the six months during which blacklisting could have affected deforestation in 2012 (see Eq 3). The second and fifth blacklists were published at the end of March 2009 and end of April 2011 and the respective treatments are set to 0.25 and 0.17. The 7th blacklist was published in October 2012 and thus is outside our analytical timeframe. Six districts were released from the blacklist during our time period. We do not expect the blacklisting effect on deforestation to vanish immediately after a district has been released from the list. Especially the second and third of the three potential impact channels discussed above are likely to result in longer term effects on deforestation dynamics. Moreover, off-listing is conditioned on having at least 80% of the eligible land registered under the CAR—a measure that effectively improves the government’s ability to monitor land cover change in the long run, also after a district was released from the list. Our treatment variable is thus coded as follows:

$$B_{it}=\{\begin{array}{ccc} \left[0,1\right] & & \text{in 1st year of blacklisting}\\ 1 & & \text{in 2nd and all subsequent years after blacklisting}\\ 0 & & \text{otherwise} \end{array}$$ (3)

i.e., treatment is coded between 0 and 1 when blacklisting occurs after the start date of the period over which deforestation is measured (August 1^st^–July 31^st^).

Confounding factors that could affect deforestation are considered in the covariates vectors *X*_*it*_ and *Z*_*i*_ of Eq 2. Our choice of covariates is based on previous empirical work on tropical deforestation in the Amazon region and beyond [3, 26–31].

Among time-invariant covariates, we consider various measures of deforestation and forest cover up until before the first blacklist in 2008 and control for district size and population density. Moreover, we control for farm characteristics, indicators of agricultural intensification and average land values, which have shown to be important predictors of deforestation in previous studies [26, 32]. Initial forest cover and average travel distance are only used in matching but omitted in regression analyses to avoid multi-collinearity. Since clouds represent a significant source of measurement error in remotely sensed deforestation data, we include cloud cover over remaining forests in all regression analysis.

Among time varying predictors, we consider GDP per capita, timber and soy prices (zero in districts without soy production) (see also [3]), and the area of settlements, protected areas, and indigenous territories in each district. All these tenure categories have been found to affect deforestation rates in previous studies [6, 33]. In addition, we control for political factors by introducing dummy variables indicating whether districts are governed by the same political party as the president of Brazil (Brazilian Social Democracy Party in 2002 and Brazilian Workers Party from 2003 to 2012). In causal mechanism analyses, we consider yearly data on the number of field-based inspections registered by the environmental protection agency, the percentage of land coverage of CAR registrations and the annual rural credit issued by the Brazilian Central Bank (BCB).

Since the group of potential control districts is likely to exhibit lower pre-treatment levels in deforestation than the treated districts, we rely on the double difference method to estimate the treatment effect of blacklisting [3, 20]. A critical assumption of the double difference method is that treated and control observations exhibit parallel time trends in the outcome variable (time-invariant heterogeneity). In other words, in the absence of blacklisting, we assume that treated and control districts would have had the same change in deforestation over time even though they exhibit different absolute levels in forest loss.

**Fig 2:**
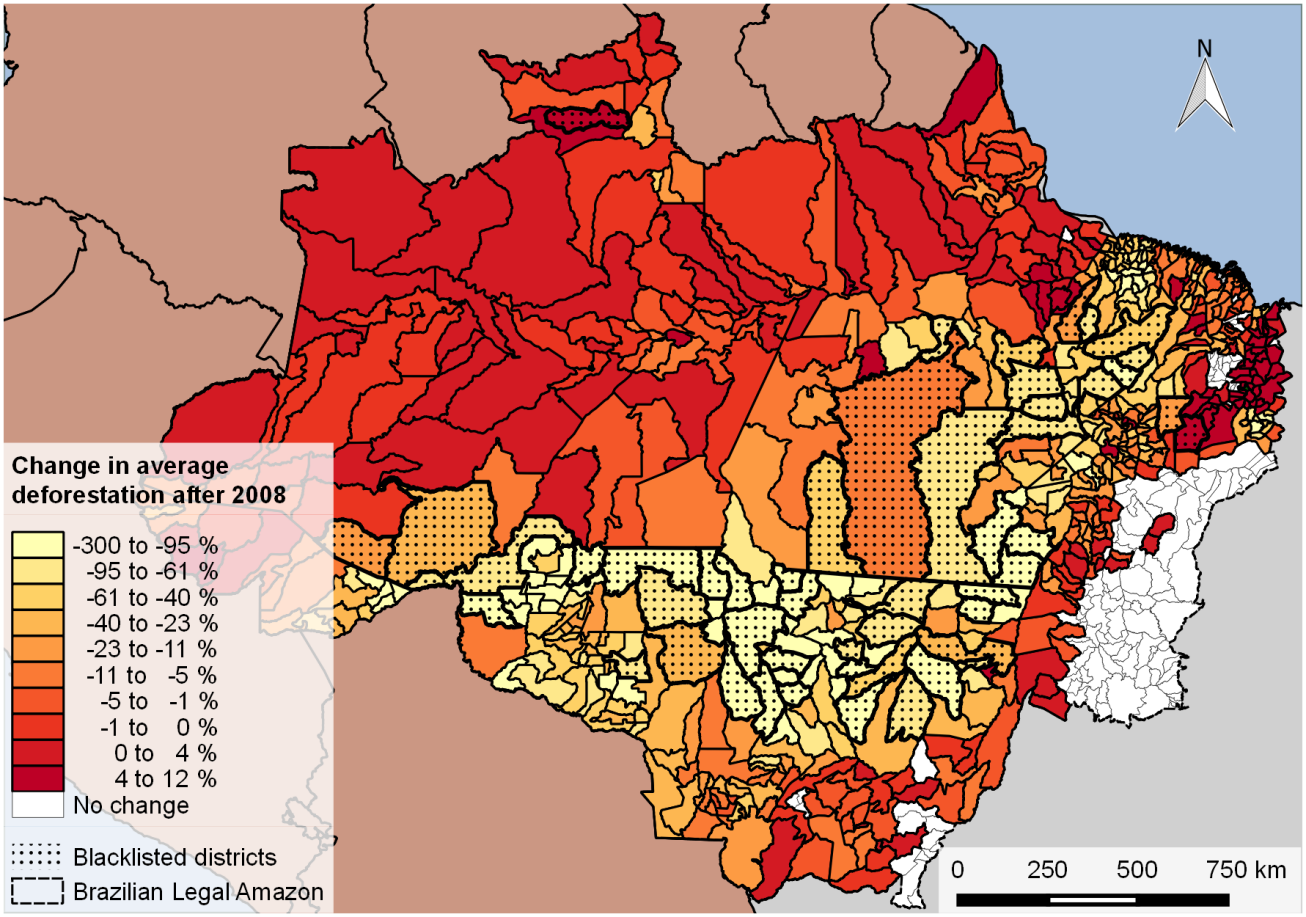
Average change in deforestation after 2008. Deforestation is measured in percentage deforestation over the district area. The change refers to the average difference between the time periods 2003–2007 and 2008 to 2012.

To ensure pre-treatment parallel time trends between control districts and blacklisted districts and to also cope with selection bias of the policy we rely on matching to filter out inappropriate controls. Matching is a frequently used quasi-experimental evaluation technique in the presence of unknown selection mechanisms [22, 34–37]. Matching relies on propensity scores or other distance measures that are derived from observed characteristics of treated and non-treated observations (here districts). Treated observations are paired with “similar” non-treated (or control) observations to reduce the bias in treatment effect estimations. A strong assumption of the matching estimator is unconfoundedness, i.e., one assumes that no other than the observed criteria were relevant in selecting districts into the blacklist. Moreover, matching requires that there is a considerable region of overlap in the distance measures or propensity scores of treated and non-treated observations of the sample. While we are able to control for a large number of potential selection criteria (see below), our sample of non-blacklisted districts is unlikely to be a satisfactory pool of potential controls because most blacklisted districts have indeed been amongst the highest deforesting districts in the Brazilian Amazon region before the blacklist was enacted (see Fig 2). Matching can help us to identify similar control observations and thus represents a sensible preprocessing step in our evaluation strategy [22]. We use 1 to 1 matching on the covariate distance between districts weighted by the inverse-variance with replacement. As described above we cannot explicitly know how the selection of blacklisted districts took place. In addition to the three official selection criteria we thus also rely on pre-treatment district characteristics as matching covariates.

The official blacklisting criteria are defined as accumulated deforested area in 2007, deforested area in 2005, 2006 and 2007 and the number of times deforestation increased over the past five years. We further use district size, the remaining forest cover as a percentage of a district area in 2007, and the average distance to the district capital estimated by Nelson [38] to account for deforestation potential and accessibility. To control for socio-economic factors we include population density in 2007 and construct indices for farm density, the share of small farms, percentage of land holders with legal land titles, the share of farm area within a district, and cattle stocking rates from the 2006 Agricultural Census. From the same source we calculated the average land value per hectare and the number of tractors per farm to control for conservation opportunity costs and capitalization levels. Further we control for GDP per capita in 2005, 2006 and 2007. To capture potential political selection determinants we also use the dummy on district mayors’ political affiliations in 2007 as explained above.

Details on the approaches used to analyze, dynamic treatment effects, spatial spillovers and causal mechanism effects are provided in the respective subsections.

## Study Area and Data

Our study area is located in the Legal Brazilian Amazon, an area of approximately five million square kilometers that extends into nine Brazilian states. Fig 2 depicts the study area highlighting changes in average deforestation in blacklisted and non-blacklisted districts after the cut-off point in 2008, when the Decree 6.321 was enacted.

From Fig 2 it becomes clear that the blacklisted districts have experienced the largest reductions in annual deforestation from the period 2003–2007 to the period 2008–2012. Large increases in average deforestation almost exclusively occurred in non-blacklisted districts, but many also experienced reductions in forest loss.

The Brazilian Legal Amazon district database from the Brazilian Institute for Geography and Statistics (IBGE) covers 771 districts. All variables are defined according to official 2007 administrative boundaries as put forward by IBGE. To avoid bias, we exclude from our sample 273 districts (none of which was blacklisted) with less than 10% forest cover in 2002. Most of these districts are located in the Amazon/Cerrado ecotone. We further exclude six districts due to missing data. This leaves us with a database of 492 districts (see S2 Fig).

To ensure consistency with 2007 administrative boundaries and control for cloud-related measurement errors we construct our dependent variable, annual deforestation, from INPE’s (Brazilian Space Research Center) publicly available vector data set for 2012. Details on how deforestation, forest, and cloud cover are defined are provided in the supplementary file S1 Text.

S1 Table summarizes the data sources used in this study. S2 Table presents descriptive statistics for the variables used in the panel data analysis and S3 Table presents means and differences in means of matching variables.

## Results

### Descriptive analysis and baseline regressions

**Fig 3:**
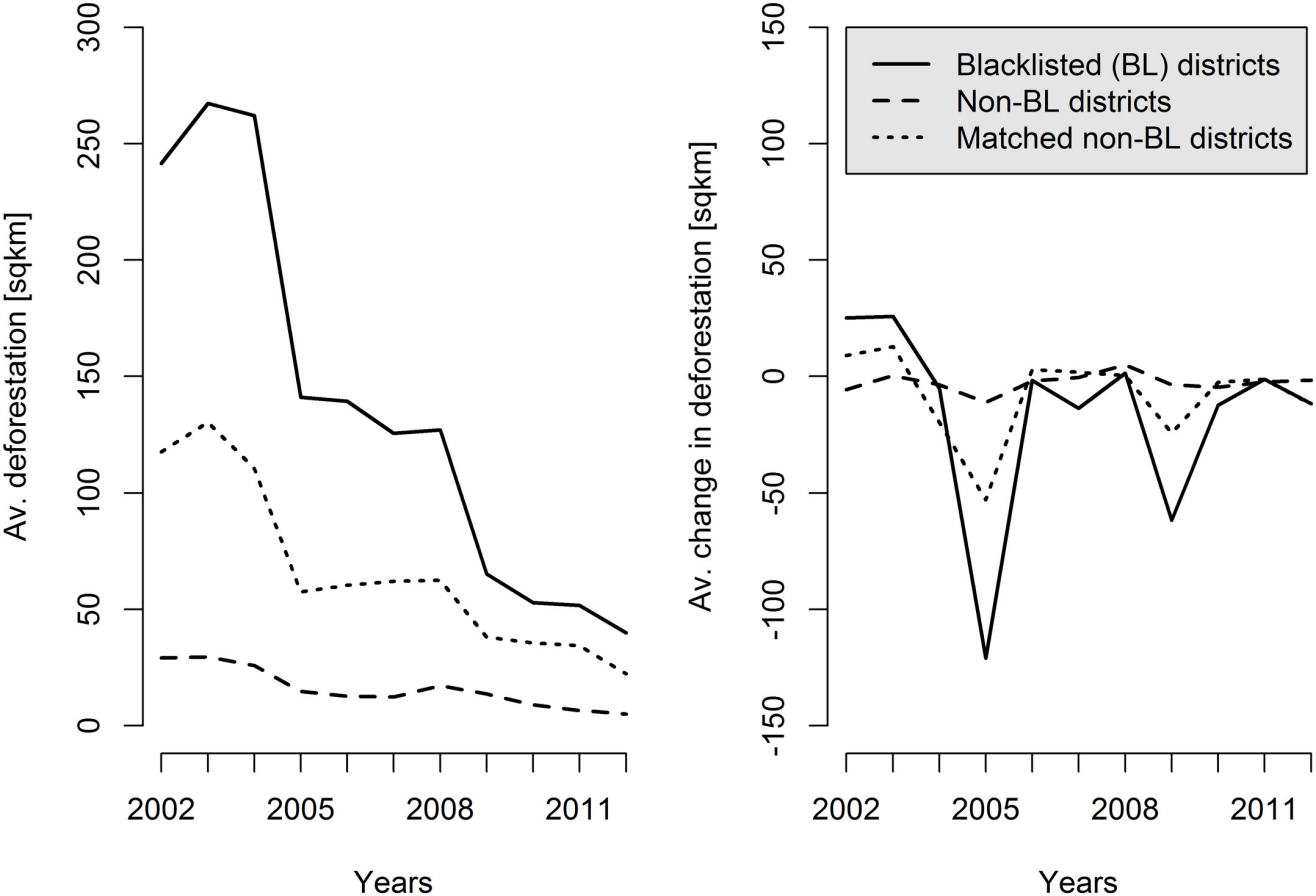
Deforestation in treatment and control districts. Average yearly deforestation levels on the left panel and average change in deforestation on the right panel. Solid lines depict averages of the blacklisted districts (50). The dashed lines show averages of all non-blacklisted districts (442). The dotted lines show averages of the matched control sample (50).

Fig 3 depicts average deforestation (left panel) and average year-to-year changes in forest loss for blacklisted and non-blacklisted districts during our study period. Average deforestation in blacklisted districts exhibits a much faster decrease than deforestation in untreated districts, but substantial decreases already occurred before the blacklist was enacted in 2008, for example between 2004 and 2005. The right panel of Fig 3 shows that average year-to-year decreases in deforestation were constantly larger in blacklisted than in control districts after 2005.

We start our analysis with all observations in a series of three baseline models using the specification in Eq 2 and gradually adding covariate groups (summary in Table 1, complete results in S4 Table). Complementary estimation results are summarized in S2 Text.

**Table 1:** Effect of blacklisting on deforestation (full sample)

Dependent	Δ ln Deforestation		
	(1)	(2)	(3)
Δ Blacklisted_it_	-0.803***	-0.992***	-0.998***
	(0.192)	(0.205)	(0.204)
Year and state effects	Yes	Yes	Yes
Time-invariant controls		Yes	Yes
Time-variant controls			Yes
Observations	4920	4920	4920
Clusters	492	492	492
Adj. R-squared	0.064	0.065	0.064

*Note*: The table reports first difference estimates with the dependent variable being the change in the log of yearly newly deforested area. Standard errors, clustered at district level, are reported in parentheses. Time-invariant and variant controls include first differences of the variables reported in S2 Table.

*** denotes significance at the 1% level.

All three models yield similar results with large and highly significant average treatment effects. The first model only includes cloud cover, year dummies and state dummies. Cloud cover is highly significant and with a coefficient close to -1. An increase in one percentage point of cloud cover is therefore associated with an almost 1% decrease in detected deforest area. The second model includes time-invariant effects of initial conditions that determine the deforestation trend in each district. Our third and preferred model includes time varying effects. Among the time-invariant covariates the share of small farmers and the cattle stocking rate, are negatively associated with increases in deforestation. Among time varying covariates, the timber price is negative and the settlement area is positively associated with deforestation. Hargrave and Kis-Katos [3] report similar results with regard to timber prices and argue that high value timber could boost long-term investment in forest and therefore contribute to lower deforestation. However, the models in Table 1 are bound to overestimate the effect of blacklisting on deforestation, because the control group contains many districts with virtually no deforestation during the observation period. We thus proceed to pre-process our data set using matching on pre-treatment characteristics as outlined earlier.

### Post-matching regressions

**Fig 4:**
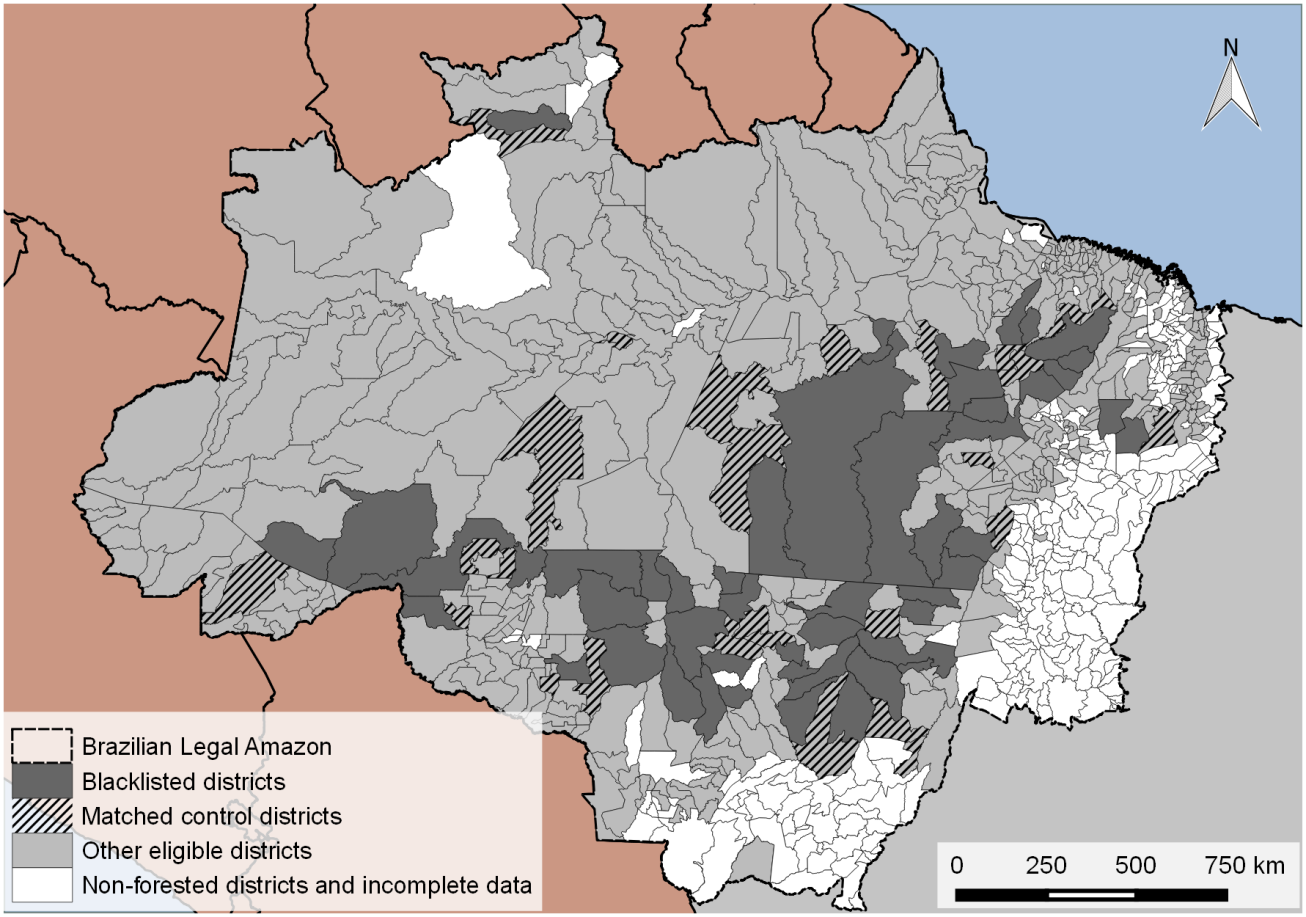
Blacklisted and matched control districts. Paired control districts are found with 1 to 1 matching with replacement using inverse-variance weights.

Matching is implemented in R using the “Matching” package [39] and the inverse-variance weights of the covariates. We find control districts by matching each blacklisted district with one non-blacklisted district using replacement. This results in 50 pairs with 50 treated districts and a set of 50 paired control observations that consist of 26 unique districts (see Fig 4). Most pairs turn out to be direct neighbors. A comparison of the covariate balance before and after matching is provided in S3 Table. For all variables, the standard mean difference has greatly improved after matching. However, significant imbalances still exist and thus a simple comparison between average deforestation in blacklisted and matched non-blacklisted groups would likely be biased. Fig 3 further compares average year-to-year changes in deforestation and deforestation trends separately for blacklisted, non-blacklisted and matched non-blacklisted districts. After matching, treated and control districts exhibit similar pre-blacklist deforestation trends (see test documentation and results in S3 Text). This gives us confidence that the critical assumption for our subsequent double difference regression is likely to hold.

We use the matched dataset to re-estimate baseline models (1–3) in Table 1. Results are presented in Table 2 (see S5 Table for complete results). Post-matching, the magnitude of the blacklisting impact on deforestation drastically decreases by more than 70% to values below 0.3. The coefficient of blacklisting in model 1 is insignificant after matching. In model 2 and 3 we include again the official selection criteria, and all matching variables. Our preferred model (3) includes all yearly information that could affect year to year changes in deforestation. After controlling for the remaining differences in the covariates the blacklisting indicator is significant. The coefficient of 0.294 suggests an average treatment effect of a 25% decrease in deforestation in blacklisted district as a result of blacklisting in each subsequent year after treatment [40].

**Table 2:** Effect of blacklisting on deforestation (matched sample).

Dependent	Δ ln Deforestation		
	(1)	(2)	(3)
Δ Blacklisted_it_	-0.249	-0.276*	-0.297*
	(0.150)	(0.153)	(0.155)
Year and state effects	Yes	Yes	Yes
Time-invariant controls		Yes	Yes
Time-variant controls			Yes
Observations	1000	1000	1000
Clusters	76	76	76
Adj. R-squared	0.251	0.245	0.258

*Note*: The table reports first difference estimates with the dependent variable being the change in the log of yearly newly deforested area. Standard errors, clustered at district level, are reported in parentheses. Time-invariant and time-variant controls include first differences of the variables reported in S2 Table. Observations are selected by a 1:1 closest neighbor matching using inverse-variance variance weights, with replacement.

* denotes significance at the 10% level.

### Dynamic treatment effects

As discussed previously, several blacklists were published over time and some districts were removed from the lists in the process. Delayed response to treatment can lead to substantial differences in treatment effects in the post-treatment periods [41]. While such differences do not change our conclusion with regard to the overall effect of blacklisting, knowledge about how the effect evolves over time (dynamic treatment effect) can be helpful for the design of a blacklisting policy. In this section we test whether the size of treatment effects varies over time. We split the original blacklisting indicator into multiple treatment indicators as follows:

$$\Delta \text{ln}D_{it}=\sum_{k=0}^{3}\Delta B\prime_{it}^{k}\beta_{k}+\Delta X\prime_{it}\gamma +Z\prime_{i}\delta +\Delta \eta_{t}+\kappa_{s}+\Delta u_{it}$$ (4)

$B_{it}^{k}$ is based on the original blacklist dummy of equation Eq 2, with the difference that $B_{it}^{k}$ overtakes only the value in year *k* after the treatment year and stays 0 otherwise. Thereby we split the original effect into 4 components. $B_{it}^{0}$ is between 0 and 1 as for the year of blacklisting and zero for all subsequent years. The treatment variables $B_{it}^{1}$,$B_{it}^{2}$ and $B_{it}^{3}$ are set to one only in the first, second and third year after blacklisting respectively, for each blacklisted district. We thereby capture the effect of blacklisting over the years. The treatment coefficients *β*_0_ to *β*_3_ can be interpreted as the average effect of blacklisting on deforestation for the respective year after blacklisting. Results are shown in Table 3. All models (1–3) produce negative and insignificant estimates for blacklisting in its initial year, but significant effects in the first and second year after treatment. For the initial year, the treatment variable is set to have only a partial effect on the overall deforestation within a district. The blacklisting effect of the initial year is thus insignificant as the change in the treatment variable is small in the beginning. Further, when blacklisting started after the dry-season, we would expect to find no effect in the first year if deforestation mainly occurs during the dry-season. The second year effects show twice as large coefficients as the first year effects. This indicates that blacklisting effects have materialized only slowly over time, which may be attributed to the gradual roll out of external support measures in the field.

**Table 3:** Dynamic effects of blacklisting.

Dependent	Δ ln Deforestation		
	(1)	(2)	(3)
Δ Blacklist effect in t	-0.399	-0.399	-0.372
	(0.314)	(0.316)	(0.325)
Δ Blacklist effect in t+1	-0.212*	-0.212*	-0.230*
	(0.123)	(0.124)	(0.126)
Δ Blacklist effect in t+2	-0.482***	-0.482***	-0.461***
	(0.156)	(0.158)	(0.156)
Δ Blacklist effect in t+3	-0.291*	-0.291*	-0.264
	(0.159)	(0.161)	(0.160)
Year and state effects	Yes	Yes	Yes
Time-invariant controls		Yes	Yes
Time-variant controls			Yes
Observations	1000	1000	1000
Clusters	76	76	76
Adj. R-squared	0.258	0.253	0.264

*Note*: The table reports first difference estimates with the dependent variable being the change in the log of yearly newly deforested area. Standard errors, clustered at district level, are reported in parentheses. Time-invariant and time-variant controls include first differences of the variables reported in S2 Table. Observations are selected by a 1:1 closest neighbor matching using inverse-variance variance weights, with replacement.

*,*** denote significance at the 10/1% level.

### Spatial spillover effects

Spatial spillover effects, such as leakage or deterrence, could bias our treatment effect estimation. In our sample, 132 out of the 442 non-blacklisted districts share at least one point on their border with another blacklisted district, i.e., they are direct neighbors. Leakage could take place if the blacklist encouraged deforestation agents to move to neighboring non-blacklisted districts. Yet, it is also possible that having a blacklisted neighboring district deters land users in non-blacklisted districts from deforesting. In the case of leakage from blacklisted to neighboring non-blacklisted districts we would overestimate the effect of blacklisting on deforestation as 46 out of the 50 matched control districts are direct neighbors. If deterrence effects of blacklisting were leading to more conservation in neighboring districts, we would underestimate the effect of blacklisting both in blacklisted districts and at the regional scale.

To test for spillover effects one approach is to include an additional dummy in Eq 2 which indicates whether a district has a neighboring district that is treated at a given point in time. However, neighbors of blacklisted districts are subject to the same selection bias as blacklisted districts. Moreover, such an approach would rely on merely four control districts in our matched dataset, which do not have a blacklisted neighbor.

Instead, we analyze spillover effects by excluding all blacklisted districts from the sample and interpreting non-blacklisted neighbors (*NB*_*i*_) of blacklisted districts as if treated. We rerun our matching analysis and conduct the post-matching regression by estimating:

$$\Delta \text{ln}D_{it|B=0}=\varphi \Delta NB_{it}+\Delta X\prime_{it}\gamma +Z\prime_{i}\delta +\Delta \eta_{t}+\kappa_{s}+\Delta u_{it}$$ (5)

Our interest lies in the effect of blacklisting on neighboring districts that have not been blacklisted, *φ*. The neighbor effect *NB*_*it*_ is set equal to one when it has at least one blacklisted direct neighbor in a given year, otherwise it equals zero. Table 4 reports the results for model specifications (1–3) as in the previous section.

**Table 4:** Spatial spillover effects of blacklisting.

Dependent	Δ ln Deforestation		
	(1)	(2)	(3)
Δ Neighbor of Blacklisted_it_	-0.159	-0.158	-0.148
	(0.106)	(0.106)	(0.108)
Year and state effects	Yes	Yes	Yes
Time-invariant controls		Yes	Yes
Time-variant controls			Yes
Observations	2640	2640	2640
Clusters	201	201	201
Adj. R-squared	0.080	0.081	0.081

*Note*: The table reports first difference estimates with the dependent variable being the change in the log of yearly newly deforested area. Standard errors, clustered at district level, are reported in parentheses. Time-invariant and time-variant controls include first differences of the variables reported in S2 Table. Observations are selected by a 1:1 closest neighbor matching using inverse-variance variance weights, with replacement. Estimated coefficients have p-values larger than 0.1.

In all 3 specifications the coefficient of the treatment indicator is negative but insignificant. The treatment effects estimated in Tables 2 and 3 are thus unlikely to be biased by spatial spillover effects from blacklisted to non-blacklisted neighboring districts.

### Robustness

In the previous sections we have estimated the impact of the blacklisting policy on deforestation. A comparison of the results in Tables 1 and 2 suggests that we would have overestimated the blacklisting effect without matching as a preprocessing step. Here we evaluate the robustness of our findings vis-à-vis alternative matching techniques and run a series of placebo tests to gain confidence in our estimated treatment effects.

We first test whether our results are robust to the use of alternative matching techniques. We compare the results from our preferred matching approach to (1), a one-to-one matching on the Mahalanobis distance, (2) a one-to-one matching on propensity scores, (3) a one-to-two matching using the inverse-variance weights, and (4) a one-to-one matching with the inverse-variance weights, but using only the three official blacklisting criteria provided in Decree 6.321. Results are presented in S6 Table. The blacklisting effect is robust in all specifications. All impact estimates are highly significant and larger than in our preferred estimation method. Our preferred method, the one-to-one matching with replacement on the covariate distance, weighted by the inverse-variance, based on an extended set of controls turns out to be the most conservative version to estimate the effect of blacklisting.

Secondly, we run a placebo analysis where we assume that blacklisting started prior to the actual start date. We shift the start date of the blacklisting policy successively to one, two, and three years before actual treatment. Results are shown in S7 Table. Column 4 repeats the main results of column 3 in Table 2. In column 3 we estimate the effect of blacklisting had it occurred in the previous year. Columns 2 and 1 show results of shifting treatment two and three years back, respectively. As expected, none of the placebo treatments are significant; indicating that the treatment effect identified earlier (Table 2) is not merely a result of preexisting differences between treated and control districts.

The results above make us confident in interpreting the observed estimates of Table 2 as a causal effect of the blacklisting policy on deforestation.

### The net treatment effect of blacklisting

Above we have produced evidence that blacklisting has led to a significant reduction in deforestation after 2007 on top of the existing decreasing trend. However, the analysis so far does not allow for conclusions with respect to the causal channels involved. In section 1 we have discussed potential impact channels that could have played a role in reinforcing the effectiveness of blacklisting: (1) Administrative disincentives, (2) reputational risk, and (3) external support/pressure. Given district level information about changes in indicators of these channels, we can empirically assess their role as causal mechanisms behind the conservation effect of the blacklist [23].

Here we follow the approach used by Ferraro and Hanauer [23] to identify the net treatment effect of blacklisting, i.e., the effect of blacklisting in the absence of effects via selected mechanisms. We consider three causal mechanisms measured annually and at district level, (1) the amount of documented environmental fines issued by the Federal Environmental Protection Agency (IBAMA), (2) the percentage of eligible area under CAR registration, and (3) the amount of official rural credit flows. We thus hypothesize that blacklisting has affected deforestation by boosting field-based enforcement action, motivating farmers to register under the CAR system, and restricting access to public rural credit. We have measured the joint effect of all potential mechanism by estimating the average treatment effect (ATT) in Table 2. The effect that is caused by blacklist-induced changes in the three above-mentioned mechanisms is called the Mechanism Average Treatment effect on the Treated (MATT). The sum of all remaining mechanism effects is the Net Average Treatment effect on the Treated (NATT). The ATT is thus composed of MATT and NATT. Our main interest in this analysis is to find the NATT that remains after controlling for the MATT. The conventional approach to control for mechanism effects is to include the variables into Eq 5 as explanatory factors. We follow this procedure in a first step and estimate the following model where *M*_*it*_ represents the mechanism values of environmental fines, CAR registrations, and public rural credit for district *i* in year *t*.

$$\Delta \text{ln}D_{it}=\beta \Delta B_{it}+\Delta X\prime_{it}\gamma +Z\prime_{i}\delta +\Delta M\prime_{it}\lambda +\eta_{t}+\kappa_{s}+\varepsilon_{it}$$ (6)

While we control for annual changes in the mechanisms in Eq 6, our estimator may still be biased if these mechanisms were actually affected by blacklisting [42]. To avoid this bias and separate the NATT from the ATT we need an empirical approach that allows us to determine (a) what the level of our mechanism indicators would have been in the absence of blacklisting, and (b) what the effect of blacklisting would have been, had the blacklist not affected the mechanisms. Based on a method proposed by Flores and Flores-Lagunes [43], Ferraro and Hanauer [23] have recently addressed similar questions in the context of protected areas.

Beyond the assumptions made up to this point, two additional assumptions are necessary to estimate the MATT and NATT [23]: (1) Selection has not been influenced by expectations that blacklisting will shift the mechanisms. In our setting, this means that conditional on observed blacklisting criteria, the possibility of increased density of field inspections, higher efforts in registering landholdings into CAR, and tighter restrictions on public credit does not affect the selection into the blacklist. Note that this assumption is is different from the expectation that the blacklisting policy as such may alter these mechanisms. (2) Changes in the mechanisms have the same effect on deforestation in districts where blacklisting has affected the mechanisms and in districts where it has not affected the mechanisms. The second assumption could theoretically be violated, for example, if field inspections in blacklisted districts would somehow have been of a different nature than inspections in other districts. To gauge the potential mechanism effects, we estimate the NATT with the mechanism effect blocked, i.e., setting field inspections, CAR registrations, and public rural credit of blacklisted districts to their counterfactual levels. The difference between the overall average treatment effect measured in Table 2 and the NATT is the joint effect of the three mechanisms.

The implementation involves three steps (note that we use a hat to indicate estimated coefficients and a tilde to indicate predicted values):

1. **Estimating counterfactual mechanism values**. Closely following Ferraro and Hanauer [23] we use two alternative approaches to estimate counterfactual mechanism values. (a) replacing the real values of the mechanisms after treatment with the values of the paired matched control districts (paired values). (b) using the matched control sample and estimate the influence of our covariate set on each mechanism (estimated values, see Eq 7 below).
  $$\Delta M_{it|B_{i}=0}=\Delta X\prime_{it}\gamma^{1}+Z\prime_{i}\delta^{1}+\Delta \eta_{t}^{1}+\kappa_{s}^{1}+\Delta u_{it}$$ (7)
  We predict counterfactual mechanism values (${\tilde{M}}_{it}$) with the point estimates ($\hat{\gamma }$,$\hat{\delta }$) for blacklisted districts, but only for the years after blacklisting had started. Therefore the new predicted mechanism vector has the properties: $\left({\tilde{M}}_{it|}|B_{it}=0)=(M_{it}|B_{it}=0\right)$. This step creates mechanism values that would have been realized in blacklisted districts if blacklisting had not influenced the mechanism policies.
2. **Counterfactual deforestation values**. Under the second assumption, we estimate how much deforestation would have occurred in blacklisted districts if blacklisting had not influenced mechanisms. We first estimate the influence of the real mechanism values on the subsample of the blacklisted districts after blacklisting as follows:
  $$\Delta \text{ln}D_{it|B_{it}=1}=\Delta X\prime_{it}\gamma^{2}+Z\prime_{i}\delta^{2}+\Delta M\prime_{it}\lambda +\Delta \eta_{t}^{2}+\kappa_{s}^{2}+\Delta u_{it}$$ (8)
  With the point estimates (${\hat{\gamma }}^{2},{\hat{\delta }}^{2},{\hat{\lambda }}^{}$) and the counterfactual mechanism values (${\tilde{M}}_{it}$) we predict the counterfactual deforestation levels (${\tilde{D}}_{it}$) for the blacklisted districts after blacklisting. Under the assumptions made above, the counterfactual deforestation represents the level of deforestation had there been no change in field inspections, CAR registrations and public rural credits as a result of blacklisting.
3. **Estimating the NATT involves** re-estimating model (3) from Table 2 using the counterfactual deforestation levels estimated in the previous step as follows:
  $$\Delta \text{ln}{\tilde{D}}_{it}=\beta \Delta B_{it}+\Delta X\prime_{it}\gamma^{3}+Z\prime_{i}\delta^{3}+\Delta \eta_{t}^{3}+\kappa_{s}^{3}+\Delta u_{it}$$ (9)

After blocking the influence of blacklisting on the mechanisms, we expect to find counterfactual levels with decreased fines, lower CAR registrations and higher amounts of public credit, relative to the observed (real) values. Using the counterfactual mechanism values, we expect to predict higher counterfactual deforestation rates than the observed rates of deforestation. Assuming a high share of the mechanism effects (MATT) within the overall effect of blacklisting (ATT), we expect to find lower and even insignificant estimates of the remaining NATT.

Results from estimating Eq 7 are presented in S8 Table. Statistics on the real and counterfactual values of the blacklisted districts after 2007 are presented in Table 5. The mean and standard deviation of the real mechanism values are reported in column (1). The two approaches used to determine counterfactual mechanism values do not yield fully consistent results. With paired values, we unexpectedly find counterfactual rural credit levels to be lower than with blacklisting. With estimated values, counterfactual environmental fine levels are higher than with blacklisting, contrary to our expectation. Differences vis-à-vis observed values with blacklisting are small, however, and the estimated counterfactual deforestation levels (Eq 8) are higher than observed deforestation for the blacklisted districts independent of the approach used to estimate counterfactual mechanism values.

**Table 5:** Statistics on counterfactual mechanism values.

Variable	Statistic	Observed values	Counterfactual paired values	Counterfactual estimated values
Lagged No. of environmental fines	Mean	58.15	47.73	71.16
	Sd.dev.	(70.58)	(72.85)	(117.94)
Car area coverage [%]	Mean	19.40	14.94	17.15
	Sd.dev.	(18.81)	(16.52)	(14.69)
Total rural credit [Mio. Reais]	Mean	26.61	20.21	653.20
	Sd.dev.	(29.24)	(25.92)	(6528.55)

*Note*: Statistics show observed (real) and counterfactual values estimated as described in section 5 on the blacklisted districts between the years 2008 to 2012. Paired values are adopted form the corresponding paired matched controls district of each blacklisted districts. Estimated values are based on estimations of mechanisms on the covariates.

The results of the mechanism analysis are shown in Table 6. In column 1 we repeat the result of our ATT estimation in Table 2 (column 3). In columns 2–5 we add mechanism effects as explanatory variables to the regression. In order to avoid reverse causality between deforestation and environmental fines we use lagged time values for environmental fines. Differences in the mechanism values between blacklisted and non-blacklisted matched district are small (Table 5). The mechanism coefficients thus tend to have small and insignificant coefficients. This exercise does not alter the estimate of the blacklisting effect vis-à-vis the results in Table 2. The coefficient remains at a 10% significance level with a negative estimate close to -0.3.

**Table 6:** Net average treatment effect of blacklisting.

	ATT					NATT	
Dependent	Δ ln Deforestation					Δ ln counterfactual Deforestation	
						paired	estimated
	(1)	(2)	(3)	(4)	(5)	(6)	(7)
Δ Blacklisted_it_	-0.297*	-0.299*	-0.298*	-0.297*	-0.299*	-0.224**	-0.211**
	(0.155)	(0.156)	(0.154)	(0.155)	(0.156)	(0.100)	(0.103)
Δ *ln* No. of fines_it-1_		0.003			0.003		
		(0.028)			(0.028)		
Δ CAR area cover_it_			-0.042		-0.043		
			(0.491)		(0.492)		
Δ *ln* Total rural credit_it_				0.010	0.010		
				(0.060)	(0.060)		
Year and state effects	Yes	Yes	Yes	Yes	Yes	Yes	Yes
Time-invariant controls	Yes	Yes	Yes	Yes	Yes	Yes	Yes
Time-variant controls	Yes	Yes	Yes	Yes	Yes	Yes	Yes
Observations	1000	1000	1000	1000	1000	1000	1000
Clusters	76	76	76	76	76	76	76
Adj. R-squared	0.258	0.258	0.258	0.258	0.256	0.308	0.313

*Note*: The table reports first difference estimates. Columns 1–5 use the change in log of yearly newly deforested area as the dependent variable. Columns 6 and 7 use the change in log of counterfactual deforestation in the blacklisted observations. Counterfactual deforestation in column 6 is constructed form paired control matches. Counterfactual deforestation in column 7 is estimated based on the set paired control matches. Standard errors, clustered at district level, are reported in parentheses. Time-invariant and time-variant controls include first differences of the variables reported in S2 Table. Observations are selected by a 1:1 closest neighbor matching using inverse-variance weights, with replacement.

*,** denote significance at the 10/5% level.

Columns 6 and 7 show results after using the paired and estimated mechanism counterfactuals, respectively, to estimate counterfactual deforestation. The new estimates represent the NATT of blacklisting, which is still negative and significant, but somewhat smaller than the ATT estimated in Table 2. The joint effect of the three potential causal mechanisms we studied thus seems to be relatively small compared to the other two potential impact channels (administrative disincentive and reputational risk) discussed above.

## Discussion

We have found a robust and significant negative effect of district blacklisting on deforestation. As outlined in the introduction, there are several theoretical pathways to explain this result, including administrative disincentives, reputational risks, and external support/pressure from both government and non-governmental organizations. We have implemented an innovative econometric method to block three potential causal mechanisms related mainly to the theoretical impact channel “external pressure/ support” that have shown to be relevant factors in Brazil’s overall strategy to reduce Amazon deforestation [5, 12]. Namely, environmental fines, geo-referenced land use registrations, and public credits. As causal mechanisms, however, these factors turned out to be of marginal importance in explaining deforestation reductions in the blacklisted districts. Clearly, this does not undermine the important role that any of these policy instruments plays in Brazil’s national strategy to reduce tropical deforestation. In fact, fine-based law enforcement, land registry (CAR), and rural credit policies, including the credit restriction imposed by the Brazilian Monetary Council in 2008 [12], apply to all districts in the Brazilian Amazon. They may only have played a more important role in blacklisted districts if blacklisting caused quantitative or qualitative shifts in their implementation. We emphasize that our mechanism analysis only captures quantitative changes in these policy components. Administrative disincentives and reputational risk in addition to qualitative changes in external support/pressure channels thus remain potentially effective drivers behind the effect of the Brazilian blacklist that could be explored in further research. Moreover, additional causal mechanisms may exist that we were not able to capture due to data limitations. These include, for example, increases in external pressure on embargoed producers through so called compliance commitments (TAC in its Portuguese acronym) issued by public prosecutors.

Like any quasi-experimental evaluation, our analysis remains prone to unobservable bias. We control for a wide range of factors comprising physical, socio-economic and political indicators. Due to limited common support, this bias could not be fully corrected for by matching, which is why we only rely on matching as a pre-processing technique [22]. We rely on the ‘weaker’ assumption of parallel time trends and control for all unobserved fixed effects.

The selection of districts to the blacklist is endogenously determined by deforestation which could violate the parallel time trend assumption. Without treatment, the decrease in deforestation rates of blacklisted districts could have materialized at a much slower pace than of control districts (see S5 Fig panel a). The likely bias from using inadequate controls with lower deforestation and more rapid drops deforestation rates would lead us to under rather than overestimate the conservation effect of blacklisting.

A common evaluation pitfall that would also violate the parallel time trend assumption and lead us to overestimate the blacklist effect is the “Ashenfelter’s or pre-program dip”. It can occur if selection is affected by unusual pre-program changes in the outcome variable [44]. In our case, a pre-blacklist peak in deforestation could hypothetically have resulted in a selection of districts that would have exhibited much faster decreases in deforestation—even in the absence of blacklisting—than any potential control district (see S5 Fig, panel b). While we cannot completely rule out such a phenomenon, we argue that it is unlikely to play a major role in explaining our findings. First, because we control for past increases in deforestation rates in our matching exercise and formally tested the hypothesis of equal pre-program deforestation trends in treated and control districts. Secondly, because the blacklist was enacted five years after average deforestation had peaked in the blacklisted districts (see Fig 3). In the two years prior to the publication of the blacklist, deforestation trends had instead been rather stable. And thirdly, the blacklisted districts have been leading deforestation rankings even prior to our observation period. Hence, and as supported by our placebo treatment analysis (S7 Table), the substantial drop in average forest loss in these districts after 2008 can hardly be attributed solely to normalization after an unusual peak.

We are thus confident that our analysis correctly identifies the blacklist as an environmental governance measure that made a substantial complementary contribution to bringing deforestation down in the Brazilian Amazon region.

## Conclusions

In this study we have used a quasi-experimental evaluation design to gauge the potential contribution of district blacklisting to the drop in deforestation rates in the Brazilian Amazon. Blacklisting has previously been used and studied in other environmental governance contexts, such as pollution control [15].

We find that the average effect of blacklisting on deforestation in blacklisted districts ranges between roughly 13–36% considering standard errors (see Table 2, model 3). This corresponds to an absolute reduction in deforestation of 600–6750 sqkm (4022 sqkm on average) from 2008 to 2012. This is far less than the 59511 sqkm cumulative conservation effect of improved field-based enforcement calculated by Assunção et al. [45] for the period from 2007–2011. However, it is more than the amount of avoided deforestation (2700 sqkm) that Assunção et al. [12] attribute to the credit restrictions that were enacted in 2008. Compared to Arima et al. [5] who report a range from 2,304–11,653 sqkm, our ATT and NATT estimates lie at the lower end and are thus also much more conservative than the 11,396 sqkm estimated by Assunção et al. [46]

In other words, between 2008 and 2012, the decision to bolster the Brazilian anti-deforestation campaign by district blacklisting has conserved an amount of forest cover that is almost equivalent to the current average annual forest loss, i.e., 4,848 sqkm in 2014 according to official INPE statistics.

At the federal level, the incremental administrative costs of maintaining the blacklist have probably been low given that no significant additional governance and implementation structure had to be put in place. However, the blacklist has reportedly induced a substantial amount of local level transaction costs and operational expenses by supporting NGOs and state-level government organizations. Putting a price tag on the Brazilian blacklisting experience therefore is not a straightforward exercise.

Relating to the effectiveness of blacklisting, this also depends on factors outside the control of the federal government. Here, one must also concede that the policy has shortcomings. For example, some states have not yet fully developed capacities to implement CAR registries at relevant scales. In such cases it seems unrealistic that districts can achieve the 80% CAR target to be removed from the list, which could undermine incentives to engage in alternative district-level efforts to reduce deforestation. Conversely, on the positive side, the blacklist has also inspired state-level initiatives, such as the Green Municipalities Program (MVP) in the state of Pará, to reward good forest stewardship in districts that voluntarily comply with the criteria for removal from the blacklist.

Given the scarce evidence on the effectiveness of transparency and accountability measures in conservation, our results should encourage experimentation with blacklisting as a complementary forest conservation measure. Clearly, a country’s administrative structure is likely to affect outcomes in significant ways. For example, Brazilian districts (i.e. municipalities) have much less legal autonomy in environmental policy than in the more decentralized governance structure of other tropical forest countries, such as Indonesia [47, 48]. The effectiveness of the diverse potential impact channels of blacklisting may thus differ substantially depending on the ability of local stakeholders to organize themselves towards the goal of being removed from a blacklist.

From the government’s point of view, as well as in the context of an international mechanism to Reducing Emissions from Deforestation and Degradation (REDD+), blacklisting appears to be a low-cost and no-regret option to increase compliance with existing forest law. Overall costs to land users clearly depend on the kind of action that blacklisting evokes at the local and district level.

## Supporting Information

S1 Data Supplementary Data. (ZIP)

S1 Fig Blacklisted districts and the blacklist criteria. The Venn diagram depicts the number of districts blacklisted and non-blacklisted from the first published list in 2008. Counts are based on PRODES official deforestation data. The blacklist was composed during the year 2008, therefore we consider for the first criterion the total deforested area until 2007. The first 36 districts with the highest deforested area fulfill criterion I. The first 36 districts with the highest deforested area between 2005 and 2007 fulfill the second criteria. All districts that at least show 3 years with increasing deforestation rates between 2003 and 2007 fulfill criterion III.
(TIF)

S2 Fig Forested districts of the Brazilian Legal Amazon. The map shows all districts of the Brazilian Legal Amazon, defined by INPE (771). In light grey are all districts with more than 10% forest cover in 2002 and complete information on all covariates used for the analysis (492). In dark grey are forested districts with incomplete data on the covariates (6).
(TIF)

S3 Fig Deforestation polygons to aggregate deforestation rates. Three detected deforestation polygons by satellite imagery are represented by the closed lines. The detection date of each polygon (end_date) represents the last date it could have been deforested. The first date an area could be deforested (start_date) is determined by the last satellite image that determined the polygon as forested. Annual deforestation rates are constructed by the sum of all polygons weighted by the share of the polygons’ timeframe within a given year.
(TIF)

S4 Fig Yearly total deforestation in the Brazilian Legal Amazon. The solid line shows yearly deforestation rates calculated by the INPE/PRODES project for the districts of the Brazilian Legal Amazon (771). The dashed line shows deforestation rates calculated from INPE’s shapefiles.
(TIF)

S5 Fig Parallel time trend assumption and potential biases conceptually. Panel a. shows the case of underestimating the impact due to selection bias where the real counterfactual of the blacklisted (had they not been treated) exhibits slower deforestation decreases than the used counterfactual, constructed from the control districts. Panel b. depicts the case of overestimating the impact where the real counterfactual of the treated districts would have had faster deforestation decreases than the used counterfactual (e.g., Ashenfelter’s dip).
(TIF)

S1 Table Data sources. (DOC)

S2 Table Summary statistics on regression variables. (DOC)

S3 Table Covariate balance before and after treatment. (DOC)

S4 Table Deforestation and blacklisted municipalities, full sample first difference regressions. (DOC)

S5 Table The effect of blacklisting after matching. (DOC)

S6 Table The effect of blacklisting after different matching techniques. (DOC)

S7 Table Placebo regressions on the timing of blacklisting. (DOC)

S8 Table The influence of covariates on mechanisms. (DOC)

S9 Table Test for pre-treatment parallel time trends. (DOC)

S1 Text Calculation of yearly deforestation rates per districts. (DOC)

S2 Text Supplementary estimation results. (DOC)

S3 Text Parallel time trends of deforestation before treatment. (DOC)
