# Supplementary figures and images for: Naming and Shaming for Conservation: Evidence from the Brazilian Amazon
Source: PLoS One. 2015 Sep 23;10(9):e0136402. doi: 10.1371/journal.pone.0136402 (PMC4580616; doi:10.1371/journal.pone.0136402)

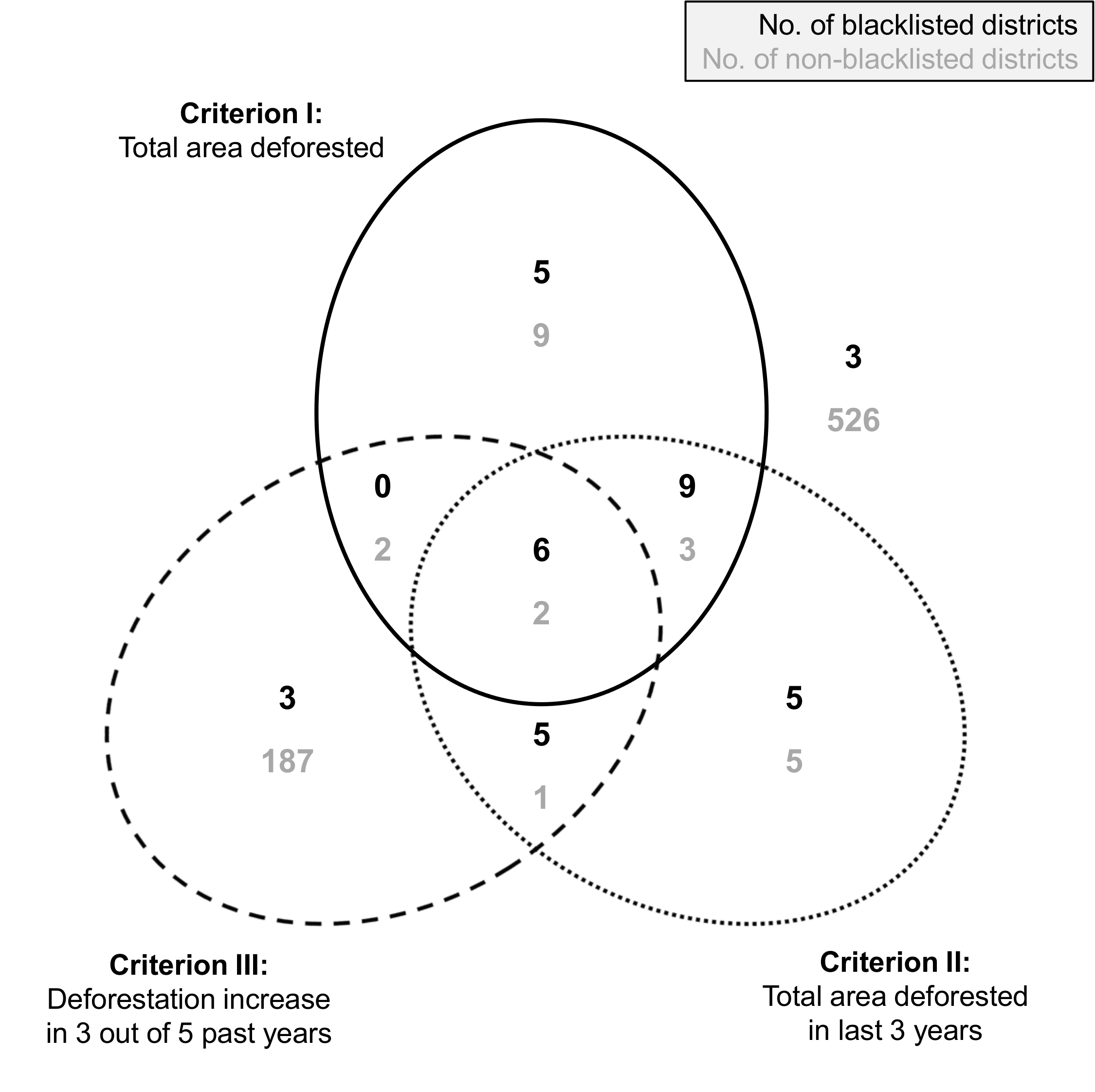

Supplement: S1 Fig — The Venn diagram depicts the number of districts blacklisted and non-blacklisted from the first published list in 2008. Counts are based on PRODES official deforestation data. The blacklist was composed during the year 2008, therefore we consider for the first criterion the total deforested area until 2007. The first 36 districts with the highest deforested area fulfill criterion I. The first 36 districts with the highest deforested area between 2005 and 2007 fulfill the second criteria. All districts that at least show 3 years with increasing deforestation rates between 2003 and 2007 fulfill criterion III. (TIF) [file pone.0136402.s002.tif]

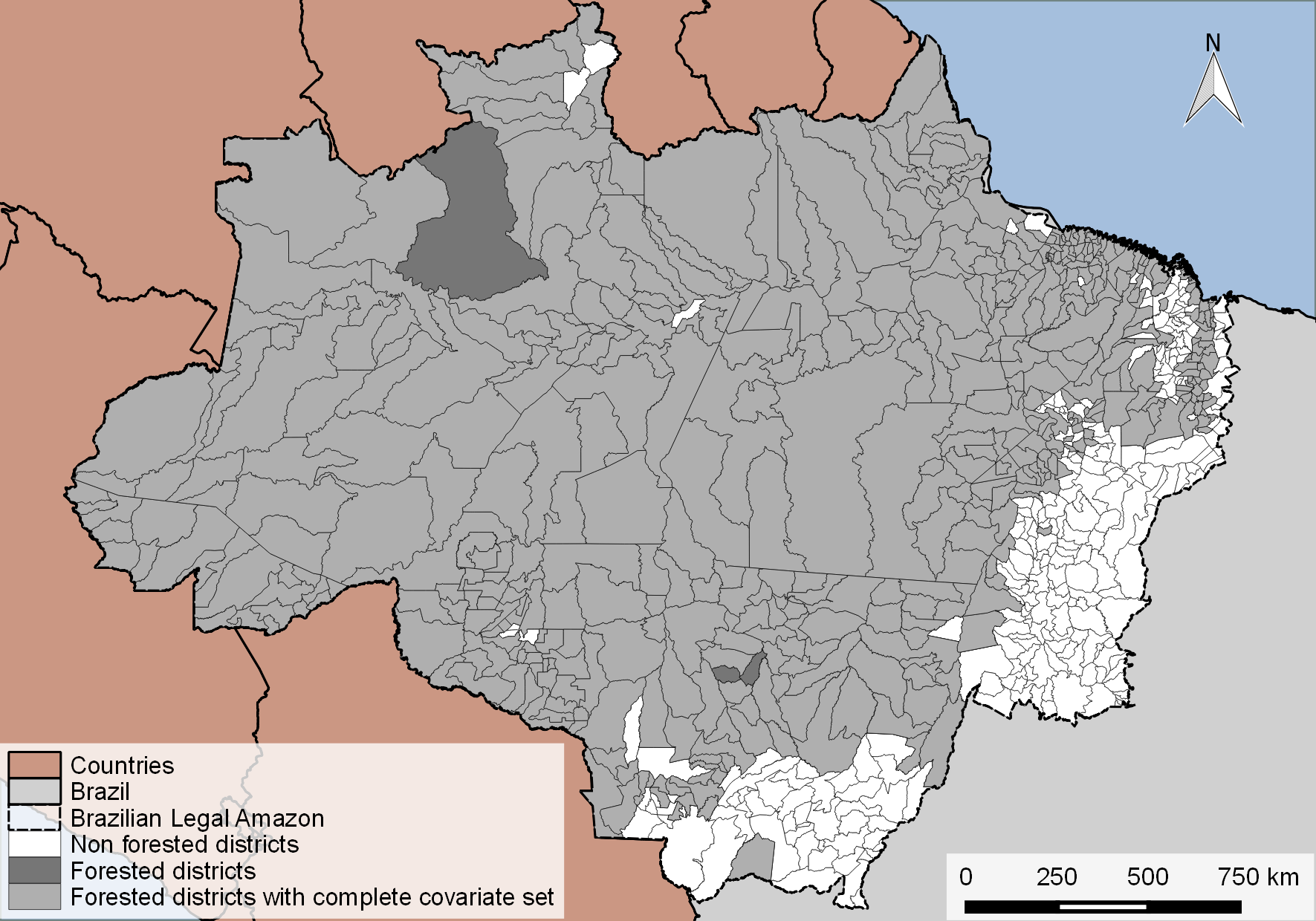

Supplement: S2 Fig — The map shows all districts of the Brazilian Legal Amazon, defined by INPE (771). In light grey are all districts with more than 10% forest cover in 2002 and complete information on all covariates used for the analysis (492). In dark grey are forested districts with incomplete data on the covariates (6). (TIF) [file pone.0136402.s003.tif]

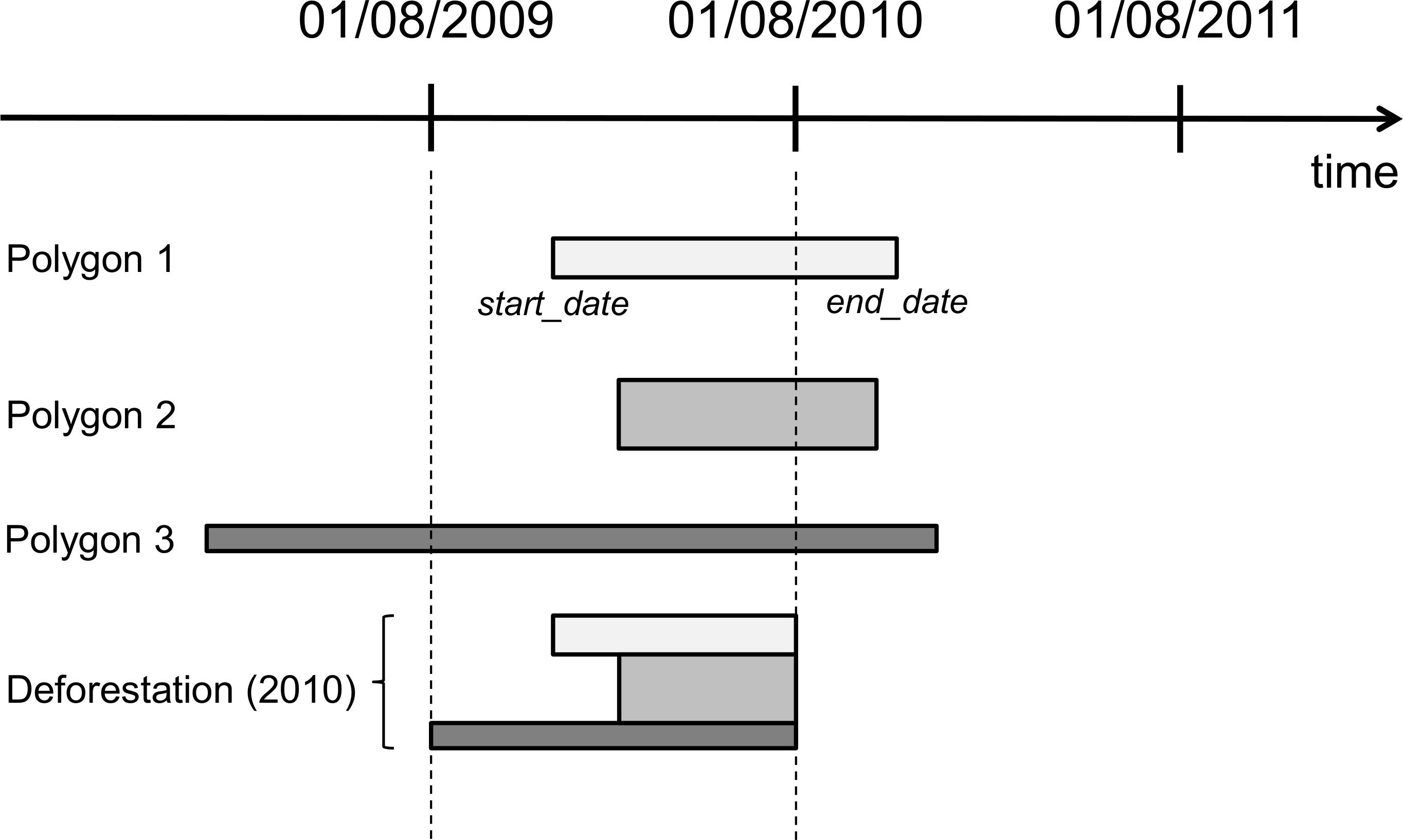

Supplement: S3 Fig — Three detected deforestation polygons by satellite imagery are represented by the closed lines. The detection date of each polygon (end_date) represents the last date it could have been deforested. The first date an area could be deforested (start_date) is determined by the last satellite image that determined the polygon as forested. Annual deforestation rates are constructed by the sum of all polygons weighted by the share of the polygons’ timeframe within a given year. (TIF) [file pone.0136402.s004.tif]

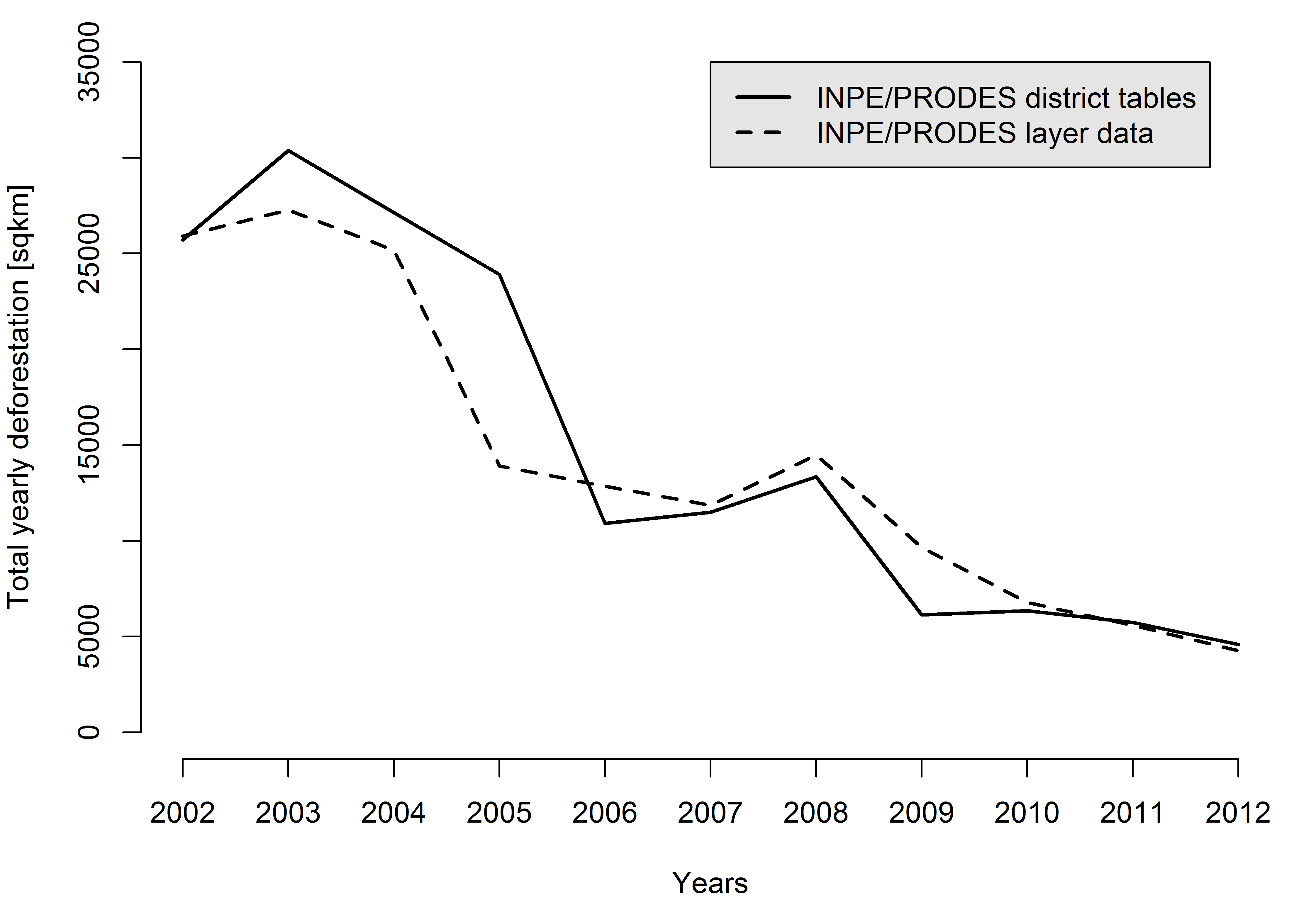

Supplement: S4 Fig — The solid line shows yearly deforestation rates calculated by the INPE/PRODES project for the districts of the Brazilian Legal Amazon (771). The dashed line shows deforestation rates calculated from INPE’s shapefiles. (TIF) [file pone.0136402.s005.tif]

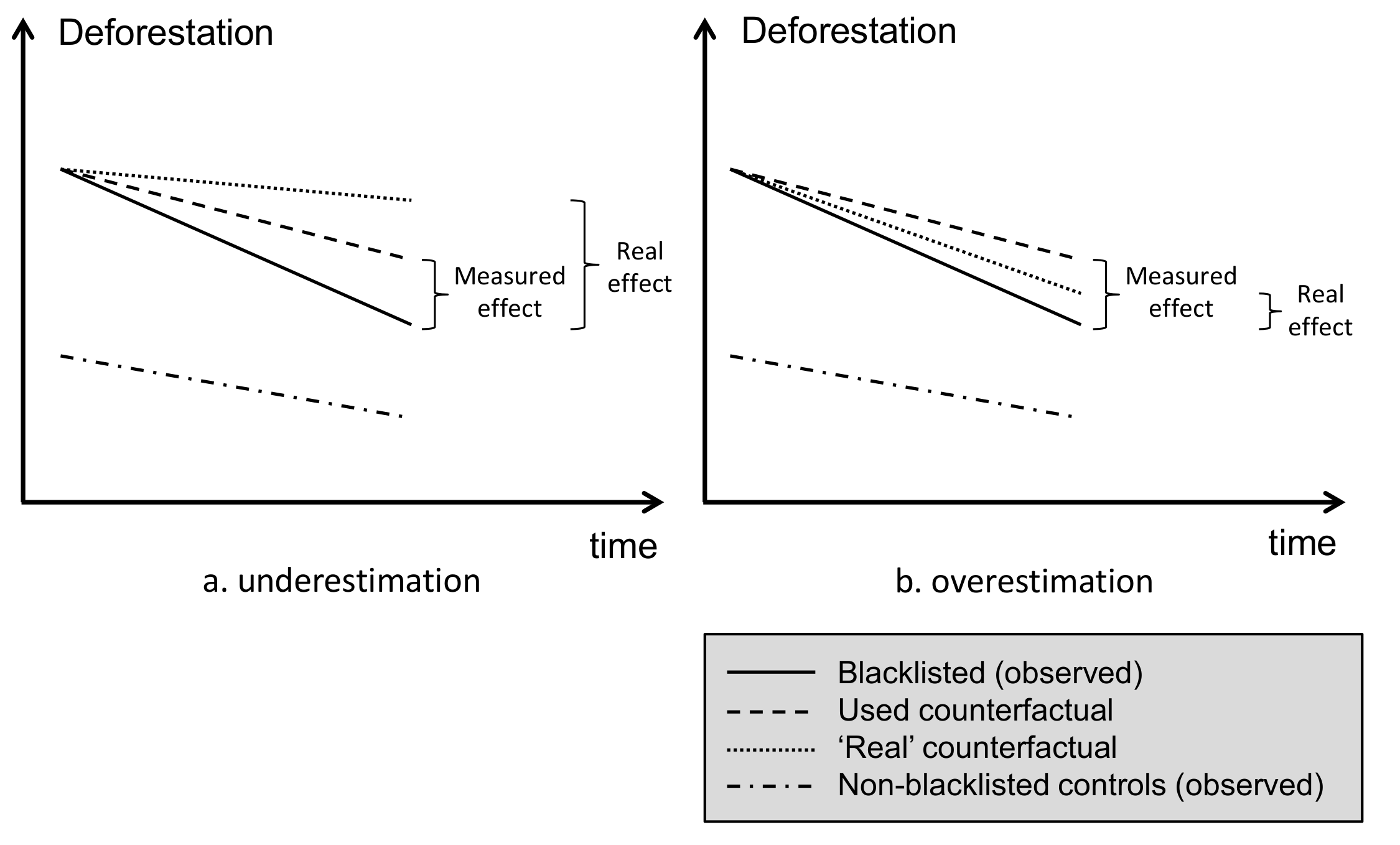

Supplement: S5 Fig — Panel a. shows the case of underestimating the impact due to selection bias where the real counterfactual of the blacklisted (had they not been treated) exhibits slower deforestation decreases than the used counterfactual, constructed from the control districts. Panel b. depicts the case of overestimating the impact where the real counterfactual of the treated districts would have had faster deforestation decreases than the used counterfactual (e.g., Ashenfelter’s dip). (TIF) [file pone.0136402.s006.tif]
