# Supplementary material for: Naming and Shaming for Conservation: Evidence from the Brazilian Amazon
Source: PLoS One. 2015 Sep 23;10(9):e0136402. doi: 10.1371/journal.pone.0136402 (PMC4580616; doi:10.1371/journal.pone.0136402)
Supplement: S1 Table — (DOC) [file pone.0136402.s007.doc]

**S1 Table. Data sources**

| **Variable** | **Year(s)** | **Source** |
| --- | --- | --- |
| Blacklist additions and removals | 2008-2012 | Decree 6.321/2007 and Provision 28/2008, Provision 102, 203/2009, Provision 66,67,68/2010 , Provision 138, 139, 175/2011, Provision 187,322,323,324/2012 |
| Deforestation and clouds | 2002-2012 | INPE-PRODES |
| Municipality list and borders | 2007 | IBGE |
| Protected areas | 2002-2012 | IBAMA |
| Indigenous areas | 2002-2012 | IBAMA |
| Settlement areas | 2002-2012 | INCRA |
| Mayors’ party affiliation | 2002-2012 | TSE |
| IPCA price deflator | 2002-2012 | IBGE |
| Soy prices | 2002-2012 | IBGE-PAM |
| Timber prices | 2002-2012 | IBGE-PEVS |
| GDP | 2002-2011 | IBGE |
| Number of farms | 2006 | IBGE Agricultural Census |
| Share of land owners | 2006 | IBGE Agricultural Census |
| Land value per ha | 2006 | IBGE Agricultural Census |
| Number of tractors | 2006 | IBGE Agricultural Census |
| Cattle stocking rate | 2006 | IBGE Agricultural Census |
| Population | 2007 | IBGE Demographic Census |
| Average distance to district center |  | Nelson |
| Field-based law enforcement inspections | 2001-2012 | IBAMA |
| Landholdings registered within the Cadastro Ambiental Rural (CAR) | 2002-2012 | Data base provided by the Amazon Environmental Research Institute (IPAM) in October 2013 |
| Rural credit | 2002-2012 | BCB |
