# Supplementary material for: Naming and Shaming for Conservation: Evidence from the Brazilian Amazon
Source: PLoS One. 2015 Sep 23;10(9):e0136402. doi: 10.1371/journal.pone.0136402 (PMC4580616; doi:10.1371/journal.pone.0136402)
Supplement: S2 Table — (DOC) [file pone.0136402.s008.doc]

**S2 Table.** Summary statistics on regression variables

|  | **N** | **Mean** | **SD** | **Min.** | **Max.** |
| --- | --- | --- | --- | --- | --- |
| **Time variant variables** | | | | | |
| Blacklisted | 5412 | 0.04 | 0.18 | 0.00 | 1.00 |
| Cloud error [share] | 5412 | 0.10 | 0.19 | 0.00 | 1.00 |
| Deforestation [sqkm] | 5412 | 28.30 | 68.82 | 0.00 | 1307.89 |
| GDP per capita [Reais] | 5412 | 9317.82 | 10439.87 | 1313.55 | 158972.89 |
| Soy price [Reais/kg] | 5412 | 0.13 | 0.28 | 0.00 | 1.99 |
| Timber price [Reais/cbm] | 5412 | 91.14 | 90.07 | 0.00 | 959.98 |
| Indigenous territory area cover [share] | 5412 | 0.08 | 0.17 | 0.00 | 1.00 |
| Multiple use protected area cover [share] | 5412 | 0.11 | 0.23 | 0.00 | 1.00 |
| Strictly protected area cover [share] | 5412 | 0.03 | 0.10 | 0.00 | 0.72 |
| Settlement area cover [share] | 5412 | 0.14 | 0.20 | 0.00 | 1.00 |
| Federal party affiliation | 5412 | 0.11 | 0.30 | 0.00 | 1.00 |
| **Time invariant variables** | | | | | |
| Initial total deforested area [sqkm] | 492 | 1087.84 | 1206.90 | 0.00 | 10253.15 |
| District area [sqkm] | 492 | 8667.78 | 15648.07 | 103.25 | 159522.59 |
| Farm area [sqkm] | 492 | 1641.25 | 2038.26 | 7.56 | 14576.02 |
| Population density [No./sqkm] | 492 | 21.30 | 97.32 | 0.09 | 1321.93 |
| Farms density [No./sqkm] | 492 | 0.59 | 0.93 | 0.00 | 12.30 |
| Share of small farms | 492 | 0.71 | 0.19 | 0.02 | 0.99 |
| No. of tractors per farm | 492 | 0.15 | 0.49 | 0.00 | 7.85 |
| Cattle rate [No./ha] | 492 | 1.50 | 2.13 | 0.00 | 31.94 |
| Share of land owners [%] | 492 | 73.34 | 23.17 | 4.49 | 100.00 |
| Land value [Reais/ha] | 492 | 1220.66 | 1007.49 | 80.00 | 7502.08 |

# Monetary figures are Million Brazilian Reais (BRL) deflated to 2012 prices, 1 BRL corresponded to USD 0.56 on average in 2012 ([**www.oanda.com**](http://www.oanda.com/)).
