# Supplementary material for: Naming and Shaming for Conservation: Evidence from the Brazilian Amazon
Source: PLoS One. 2015 Sep 23;10(9):e0136402. doi: 10.1371/journal.pone.0136402 (PMC4580616; doi:10.1371/journal.pone.0136402)
Supplement: S3 Table — (DOC) [file pone.0136402.s009.doc]

**S3 Table.** Covariate balance before and after treatment

| Covariate | Status | Mean blacklist | Mean non-blacklist | Difference in means | Normalized difference | Mean eQQ difference | % improvement mean difference |
| --- | --- | --- | --- | --- | --- | --- | --- |
| Total deforested area in 2007 | Unmatched | 3937.86 | 966.21 | 2971.65 | 1.21 | 2933.63 |  |
|  | Matched | 3937.86 | 2351.69 | 1586.17 | 0.65 | 1586.17 | 0.47 |
| Deforestation in 2005 | Unmatched | 141.02 | 14.68 | 126.35 | 1.00 | 125.10 |  |
|  | Matched | 141.02 | 57.41 | 83.62 | 0.66 | 83.62 | 0.34 |
| Deforestation in 2006 | Unmatched | 139.28 | 12.69 | 126.59 | 0.91 | 125.35 |  |
|  | Matched | 139.28 | 60.36 | 78.92 | 0.56 | 78.92 | 0.38 |
| Deforestation in 2007 | Unmatched | 125.55 | 12.28 | 113.27 | 0.75 | 111.43 |  |
|  | Matched | 125.55 | 62.11 | 63.44 | 0.42 | 63.44 | 0.44 |
| Deforestation increases | Unmatched | 2.38 | 1.70 | 0.68 | 1.07 | 0.74 |  |
|  | Matched | 2.38 | 2.44 | -0.06 | -0.09 | 0.06 | 1.09 |
| District area | Unmatched | 18106.38 | 7600.14 | 10506.24 | 0.42 | 9492.81 |  |
|  | Matched | 18106.38 | 12569.24 | 5537.14 | 0.22 | 5953.90 | 0.47 |
| Forest cover in 2007 | Unmatched | 0.58 | 0.46 | 0.12 | 0.63 | 0.16 |  |
|  | Matched | 0.58 | 0.54 | 0.04 | 0.20 | 0.07 | 0.69 |
| Population density in 2007 | Unmatched | 3.13 | 23.36 | -20.23 | -6.35 | 36.28 |  |
|  | Matched | 3.13 | 3.03 | 0.10 | 0.03 | 0.53 | 1.00 |
| Farms per sqkm in 2006 | Unmatched | 0.16 | 0.64 | -0.49 | -3.70 | 0.66 |  |
|  | Matched | 0.16 | 0.20 | -0.04 | -0.32 | 0.04 | 0.91 |
| Share of small farms in 2006 | Unmatched | 0.64 | 0.72 | -0.08 | -0.48 | 0.08 |  |
|  | Matched | 0.64 | 0.66 | -0.02 | -0.13 | 0.03 | 0.73 |
| Farm area cover in 2006 | Unmatched | 0.39 | 0.41 | -0.02 | -0.12 | 0.11 |  |
|  | Matched | 0.39 | 0.40 | -0.02 | -0.09 | 0.03 | 0.22 |
| No. of tractors per farm in 2006 | Unmatched | 0.31 | 0.14 | 0.17 | 0.37 | 0.27 |  |
|  | Matched | 0.31 | 0.19 | 0.12 | 0.25 | 0.12 | 0.32 |
| Cattle rate in 2006 | Unmatched | 1.18 | 1.54 | -0.36 | -0.86 | 0.87 |  |
|  | Matched | 1.18 | 1.26 | -0.08 | -0.19 | 0.10 | 0.78 |
| Share of land owners | Unmatched | 77.70 | 72.85 | 4.85 | 0.26 | 6.02 |  |
|  | Matched | 77.70 | 82.52 | -4.82 | -0.26 | 5.38 | 1.99 |
| Land value in 2005 | Unmatched | 1422.57 | 1197.82 | 224.74 | 0.30 | 379.43 |  |
|  | Matched | 1422.57 | 1429.67 | -7.10 | -0.01 | 184.18 | 1.03 |
| Av. distance to district center | Unmatched | 839.92 | 706.20 | 133.72 | 0.26 | 340.82 |  |
|  | Matched | 839.92 | 799.60 | 40.32 | 0.08 | 136.69 | 0.70 |
| Federal party affiliation in 2007 | Unmatched | 0.08 | 0.11 | -0.03 | -0.10 | 0.04 |  |
|  | Matched | 0.08 | 0.06 | 0.02 | 0.07 | 0.02 | 1.70 |
| GDP per capita in 2005 | Unmatched | 11381.30 | 7984.97 | 3396.33 | 0.43 | 4856.88 |  |
|  | Matched | 11381.30 | 9821.59 | 1559.71 | 0.20 | 1571.07 | 0.54 |
| GDP per capita in 2005 | Unmatched | 10131.02 | 8071.93 | 2059.09 | 0.40 | 3564.31 |  |
|  | Matched | 10131.02 | 9506.64 | 624.38 | 0.12 | 1448.08 | 0.70 |
| GDP per capita in 2005 | Unmatched | 12060.29 | 9062.95 | 2997.34 | 0.37 | 4161.11 |  |
|  | Matched | 12060.29 | 10353.12 | 1707.16 | 0.21 | 1859.55 | 0.43 |
| Indigenous territory cover | Unmatched | 0.14 | 0.07 | 0.06 | 0.37 | 0.08 |  |
|  | Matched | 0.14 | 0.11 | 0.03 | 0.15 | 0.03 | 0.60 |
| Multiple use protected area cover | Unmatched | 0.04 | 0.13 | -0.09 | -1.17 | 0.10 |  |
|  | Matched | 0.04 | 0.07 | -0.03 | -0.36 | 0.03 | 0.69 |
| Strictly protected area cover | Unmatched | 0.03 | 0.04 | -0.01 | -0.19 | 0.03 |  |
|  | Matched | 0.03 | 0.02 | 0.00 | 0.04 | 0.02 | 1.23 |
| Settlement area cover | Unmatched | 0.10 | 0.16 | -0.06 | -0.54 | 0.07 |  |
|  | Matched | 0.10 | 0.12 | -0.03 | -0.25 | 0.03 | 0.54 |
