# Supplementary material for: Naming and Shaming for Conservation: Evidence from the Brazilian Amazon
Source: PLoS One. 2015 Sep 23;10(9):e0136402. doi: 10.1371/journal.pone.0136402 (PMC4580616; doi:10.1371/journal.pone.0136402)
Supplement: S4 Table — (DOC) [file pone.0136402.s010.doc]

**S4 Table. Deforestation and blacklisted municipalities, full sample** first difference regressions

| Dependent | Δ ln Deforestation | | |
| --- | --- | --- | --- |
|  | (1) | (2) | (3) |
| Δ Blacklisted_it_ | -0.803*** | -0.992*** | -0.998*** |
|  | (0.192) | (0.205) | (0.204) |
| Δ Cloud error_it_ | -0.978*** | -0.971*** | -0.984*** |
|  | (0.171) | (0.171) | (0.171) |
| Δ *ln* Initial total deforested area_i_ |  | 0.000** | 0.000** |
|  |  | (0.000) | (0.000) |
| Δ *ln* District area_i_ |  | 0.000*** | 0.000*** |
|  |  | (0.000) | (0.000) |
| Δ *ln* Farm area_i_ |  | 0.000 | 0.000 |
|  |  | (0.000) | (0.000) |
| Δ *ln* Population density_i_ |  | -0.000 | -0.000 |
|  |  | (0.000) | (0.000) |
| Δ *ln* Farms per sqkm_i_ |  | 0.022 | 0.022 |
|  |  | (0.017) | (0.017) |
| Δ *ln* Share of small farms_i_ |  | -0.180* | -0.180* |
|  |  | (0.092) | (0.094) |
| Δ *ln* No. of tractors per farm_i_ |  | -0.085 | -0.086 |
|  |  | (0.055) | (0.056) |
| Δ *ln* Cattle rate_i_ |  | -0.026*** | -0.026*** |
|  |  | (0.009) | (0.009) |
| Δ *ln* Share of land owners_i_ |  | 0.000 | 0.000 |
|  |  | (0.001) | (0.001) |
| Δ *ln* Land value_i_ |  | -0.000 | -0.000 |
|  |  | (0.000) | (0.000) |
| Δ *ln* GDP per capita_it-1_ |  |  | -0.024 |
|  |  |  | (0.133) |
| Δ *ln* Soy price_it-1_ |  |  | 0.041 |
|  |  |  | (0.116) |
| Δ *ln* Timber price_it-1_ |  |  | -0.059** |
|  |  |  | (0.026) |
| Δ Indigenous territory area cover_it_ |  |  | 0.344 |
|  |  |  | (0.397) |
| Δ Multiple use protected area cover_it_ |  |  | -0.045 |
|  |  |  | (0.392) |
| Δ Strictly protected area cover_it_ |  |  | -0.061 |
|  |  |  | (0.280) |
| Δ Settlement cover_it_ |  |  | 0.731** |
|  |  |  | (0.342) |
| Δ Federal party affiliation_it_ |  |  | 0.007 |
|  |  |  | (0.075) |
| Constant | -0.056* | 0.093 | 0.092 |
|  | (0.034) | (0.099) | (0.104) |
| Year and state effects | Yes | Yes | Yes |
| Observations | 4920 | 4920 | 4920 |
| Clusters | 492 | 492 | 492 |
| Adj. R-squared | 0.064 | 0.065 | 0.064 |

*Note:* The table reports first difference estimates with the dependent variable being the change in the log of yearly newly deforested area. Standard errors, clustered at district level, are reported in parentheses. Observations are selected by a 1:1 closest neighbor matching using inverse-variance weights, with replacement. *,**,*** denote significance at the 10/5/1% level
