# Supplementary material for: Naming and Shaming for Conservation: Evidence from the Brazilian Amazon
Source: PLoS One. 2015 Sep 23;10(9):e0136402. doi: 10.1371/journal.pone.0136402 (PMC4580616; doi:10.1371/journal.pone.0136402)
Supplement: S6 Table — (DOC) [file pone.0136402.s012.doc]

**S6 Table. The effect of blacklisting after different matching techniques**

| Dependent | Δ ln Deforestation | | | |
| --- | --- | --- | --- | --- |
|  | 1:1 MD | 1:1 PS | 1:2 IV | 1:1 IV restricted |
|  | (1) | (2) | (3) | (4) |
| Δ Blacklisted_it_ | -0.346** | -0.444*** | -0.323** | -0.437*** |
|  | (0.148) | (0.147) | (0.142) | (0.161) |
| Year and state effects | Yes | Yes | Yes | Yes |
| Time invariant covariates | Yes | Yes | Yes | Yes |
| Time variant covariates | Yes | Yes | Yes | Yes |
| Observations | 1000 | 1200 | 2000 | 1000 |
| Clusters | 88 | 70 | 95 | 71 |
| Adj. R-squared | 0.219 | 0.340 | 0.268 | 0.255 |

*Note:* The table reports first difference estimates with the dependent variable being the change in the log of yearly newly deforested area. Standard errors, clustered at district level, are reported in parentheses. Observations of column (1) are selected by a 1:1 matching on the Mahalonobis distance. Observations of column (2) are selected by a 1:1 matching on the propensity scores. Observations of column (3) are selected by a 1:2 matching using inverse-variance weights. Observations of column (4) are selected by a 1:1 matching using inverse-variance weights based on a reduced sample of covariates (official criteria, see section 1). Time invariant and variant controls include first differences of the variables reported in Table S1.2. **,*** denote significance at the 5/1% level
