# Supplementary material for: Naming and Shaming for Conservation: Evidence from the Brazilian Amazon
Source: PLoS One. 2015 Sep 23;10(9):e0136402. doi: 10.1371/journal.pone.0136402 (PMC4580616; doi:10.1371/journal.pone.0136402)
Supplement: S7 Table — (DOC) [file pone.0136402.s013.doc]

**S7 Table. Placebo regressions on the timing of blacklisting**

| Dependent | Δ ln Deforestation | | | |
| --- | --- | --- | --- | --- |
|  | (1) | (2) | (3) | (4) |
|  | t-3 | t-2 | t-1 | t-0 |
| Δ Blacklisted_it-k_ | 0.178 | -0.035 | -0.083 | -0.297* |
|  | (0.116) | (0.145) | (0.153) | (0.155) |
| Year and state effects | Yes | Yes | Yes | Yes |
| Time invariant covariates | Yes | Yes | Yes | Yes |
| Time variant covariates | Yes | Yes | Yes | Yes |
| Observations | 1000 | 1000 | 1000 | 1000 |
| Clusters | 76 | 76 | 76 | 76 |
| Adj. R-squared | 0.258 | 0.255 | 0.256 | 0.258 |

*Note:* The table reports first difference estimates with the dependent variable being the change in the log of yearly newly deforested area. Standard errors, clustered at district level, are reported in parentheses. Observations are selected by a 1:1 closest neighbor matching using inverse-variance weights, with replacement. * denotes significance at the 10% level
