# Supplementary material for: Naming and Shaming for Conservation: Evidence from the Brazilian Amazon
Source: PLoS One. 2015 Sep 23;10(9):e0136402. doi: 10.1371/journal.pone.0136402 (PMC4580616; doi:10.1371/journal.pone.0136402)
Supplement: S8 Table — (DOC) [file pone.0136402.s014.doc]

**S8 Table. The influence of covariates on mechanisms**

| Dependent | Δ ln No of env. fines | Δ Car area coverage | Δ ln Rural credit | |
| --- | --- | --- | --- | --- |
|  | (1) | (2) | (3) | |
| Δ Cloud error_it_ | -0.730 | 0.006 | | 0.425** |
|  | (0.623) | (0.011) | | (0.162) |
| Δ *ln* Initial total deforested area_i_ | -0.000 | 0.000** | | 0.000 |
|  | (0.000) | (0.000) | | (0.000) |
| Δ *ln* District area_i_ | -0.000 | -0.000*** | | -0.000** |
|  | (0.000) | (0.000) | | (0.000) |
| Δ *ln* Farm area_i_ | 0.000 | 0.000 | | 0.000 |
|  | (0.000) | (0.000) | | (0.000) |
| Δ *ln* Population density_i_ | 0.019 | -0.000 | | 0.026 |
|  | (0.020) | (0.002) | | (0.021) |
| Δ *ln* Farms per sqkm_i_ | -0.503* | -0.044* | | -0.324 |
|  | (0.285) | (0.023) | | (0.269) |
| Δ *ln* Share of small farms_i_ | -0.335 | 0.110*** | | -0.321 |
|  | (0.359) | (0.016) | | (0.220) |
| Δ *ln* No. of tractors per farm_i_ | -0.387 | 0.033** | | -0.408** |
|  | (0.351) | (0.014) | | (0.189) |
| Δ *ln* Cattle rate_i_ | -0.030 | 0.005 | | 0.037 |
|  | (0.082) | (0.004) | | (0.035) |
| Δ *ln* Share of land owners_i_ | 0.001 | 0.000*** | | -0.004*** |
|  | (0.002) | (0.000) | | (0.001) |
| Δ *ln* Land value_i_ | -0.000 | -0.000 | | -0.000 |
|  | (0.000) | (0.000) | | (0.000) |
| Δ *ln* GDP per capita_it-1_ | -0.271 | -0.036*** | | -0.250* |
|  | (0.340) | (0.011) | | (0.140) |
| Δ *ln* Soy price_it-1_ | -0.012 | -0.006 | | 0.260* |
|  | (0.594) | (0.010) | | (0.145) |
| Δ *ln* Timber price_it-1_ | -0.079 | -0.006* | | 0.008 |
|  | (0.131) | (0.003) | | (0.018) |
| Δ Indigenous territory area cover_it_ | 1.822* | -0.084*** | | -2.316*** |
|  | (0.908) | (0.025) | | (0.405) |
| Δ Multiple use protected area cover_it_ | -0.158 | 0.033 | | 0.285 |
|  | (1.708) | (0.044) | | (0.395) |
| Δ Strictly protected area cover_it_ | -2.824 | 0.136 | | 3.527*** |
|  | (3.284) | (0.117) | | (0.862) |
| Δ Settlement cover_it_ | 0.630 | -0.020 | | -0.778*** |
|  | (0.563) | (0.029) | | (0.197) |
| Δ Federal party affiliation_it_ | 0.316 | 0.033* | | -0.140 |
|  | (0.329) | (0.017) | | (0.224) |
| Constant | 1.282*** | -0.122*** | | 1.067*** |
|  | (0.449) | (0.023) | | (0.292) |
| Year and state effects | Yes | Yes | | Yes |
| Year and state effects | Yes | Yes | Yes | |
| Time invariant controls | Yes | Yes | Yes | |
| Time variant controls | Yes | Yes | Yes | |
| Observations | 500 | 500 | 500 | |
| Clusters | 76 | 76 | 76 | |
| Adj. R-squared | 0.102 | 0.523 | 0.145 | |

*Note:* The table reports first difference. Car area coverage is measured between 0 and 1. Standard errors, clustered at district level, are reported in parentheses. Observations are selected by a 1:1 closest neighbor matching using inverse-variance weights, with replacement. *,**,*** denote significance at the 10/5/1% level
