# Supplementary material for: Naming and Shaming for Conservation: Evidence from the Brazilian Amazon
Source: PLoS One. 2015 Sep 23;10(9):e0136402. doi: 10.1371/journal.pone.0136402 (PMC4580616; doi:10.1371/journal.pone.0136402)
Supplement: S9 Table — (DOC) [file pone.0136402.s015.doc]

**S9 Table. Test for pre**-treatment parallel time trends

| Dependent | Δ ln Deforestation | | |
| --- | --- | --- | --- |
|  | (1) | (2) | (3) |
| Blacklisted_i_ | 127.197 |  |  |
|  | (78.503) |  |  |
| Year | -0.012 | -0.065*** | -0.016 |
|  | (0.033) | (0.025) | (0.026) |
| Blacklisted_i_ * Year | -0.063 |  |  |
|  | (0.039) |  |  |
| State effects | Yes | Yes | Yes |
| Time invariant controls | Yes | Yes | Yes |
| Time variant controls | Yes | Yes | Yes |
| Observations | 500 | 250 | 250 |
| Clusters | 76 | 50 | 26 |
| Adj. R-squared | 0.156 | 0.154 | 0.149 |

*Note:* The table reports first difference estimates with the dependent variable being the change in the log of yearly newly deforested area. Standard errors, clustered at district level, are reported in parentheses. Time invariant and variant controls include first differences of the variables reported in Table S1.2. Observations are selected by a 1:1 closest neighbor matching using inverse-variance weights, with replacement. *** denotes significance at the 1% level
