# Supplementary material for: Naming and Shaming for Conservation: Evidence from the Brazilian Amazon
Source: PLoS One. 2015 Sep 23;10(9):e0136402. doi: 10.1371/journal.pone.0136402 (PMC4580616; doi:10.1371/journal.pone.0136402)
Supplement: S1 Text — (DOC) [file pone.0136402.s016.doc]

# Supplementary Information

**S1 Text. Calculation of yearly deforestation rates per districts.**

**Calculation of annual deforestation.** In addition to district-level annual gross deforestation tables, the Brazilian Space Research Center (INPE) provides the spatial data files used to estimate gross deforestation rates for public download on an annual basis. The data is provided in shape file format for each Landsat path row. In the 228 downloadable layers that cover the Brazilian Legal Amazon (BLA) in 2012, polygons are classified into remaining forest, hydrography, non-forest (swamps, savanna), clouds, residual, deforested and detected in 1997, deforested and detected in 1999, deforested and detected in each year from 2000 to 2012. INPES classification methodology of deforestation detects only clear cut deforestation on areas that have never been deforested. Forest regrowth on abandoned plots is not accounted for. For each deforested polygon a detection date is provided which we call the *end_date*. To construct annual deforestation levels from the spatial data we need to know when the deforested polygon had last been classified as forest, we call this the *start_date*. For each deforestation polygon INPE also reports the number of years it was covered by clouds during previous years. To define the start date for each polygon we thus rely on the shape files provided for previous years. In S3 Fig. polygons with different start and end dates are shown schematically. Each of the three polygons could have been deforested at any time or gradually between its start and end date. Polygon 3, for example, spreads over 2 years, as it has been covered by clouds in the image of 2009. We assume that the polygon could have been deforested with the same probability at each point in the relevant time frame and then aggregate the expected deforestation for each day to estimate how much deforestation has most likely occurred between the 1^st^ of August and the 31^st^ of July of each year in each district. This procedure differs slightly from INPE’s methodology described in Camara et al. . Here it is assumed that deforestation could only have occurred in a specific dry season for each pathrow.

The main difference between our and INPE’s approach is that we do not calculate expected deforestation below clouds. INPE uses the cloud coverage of each year to estimate deforestation below clouds relative to the ratio of deforestation to forest within a specific area. The published deforestation rates therefore refer to detected deforestation plus expected deforestation below clouds. For the purpose of estimating the impact of blacklisting at a specific date on deforestation after this date, we prefer to use raw deforestation calculated as described above. We then use cloud cover (see below) as an indicator of measurement error in our regression. By using the official 2007 administrative boundary shape file for Brazil as published by the Brazilian Institute for Geography and Statistics (IBGE) we ensure that our deforestation estimate is consistent across all data sources.

S4 Fig. depicts the total aggregated yearly deforestation rates per district published by INPE and our estimation of deforestation rates. The data processing and area calculations are conducted with PostgreSQL 9.2.3 and the PostGIS 2.0.1 add-on. The difference between our calculated total deforestation in the BLA and the official values are the result of large cloud cover in the early years of the observation period.

**Calculation of yearly forest area.** Our forest area calculations for each year are anchored to the year 2012 based on the shape files published in the same year. We calculate the remaining forest area in 2012. INPE only classifies cloud coverage above the remaining forest areas. We therefore add to the calculated forest area in 2012 area of clouds. Thereafter we subtract from our calculated yearly deforestation rates (see above) to arrive at forest area for each year prior to 2012. As a result forest, non-forested and deforested areas always sum up to 100% of the district area.

**Calculation of measurement (cloud) errors.** Since clouds are not randomly distributed over space and time we have to control for the fact that some deforestation polygons were detected only after the respective area had been under clouds for several years. Especially in the most recent year of our time period the cloud cover will veil some deforestation. But also between 2000-2005 PRODES estimates suffered from large cloud cover. We use the percentage share of yearly cloud areas over the remaining forest area as a yearly indicator to the measurement error of deforestation within districts.

**Area calculation of protected areas.** Spatial information on the protected areas of Brazil from IBAMA as reported in S1 Table below. We calculate protected area coverage within the districts of the BLA separately for Multiple Use Reserves, Strictly Protected Reserves and Indigenous Areas. The information on each protected unit comes with its respective decree number and date of establishments. This allows us to calculate the yearly cover of protected areas within each district for each year. We use the same timeframe that applies to our deforestation data from August to July. For example a protected area that was established before the 31st of July 2010 will be used to calculate total protected area per district in 2010.

**Area calculation of landholdings registered within the Cadastro Ambiental Rural (CAR).** The spatial information on each registered landholding is divided into definite and provisionary CAR. The former comprises CAR properties that have already gone through the verification process of the States’ Special Secretariat of Environment (SEMA). Because the relative share of definite CARs is rather small we rely on the total registered area. Every CAR registration comes with a date and/or a date of submission. We rely on this information to calculate the total annual CAR area within districts using July 31^st^ as cut-off date. The spatial database on CAR registrations has a large amount of overlaying polygons. We deal with this fact by merging all CAR registrations within each year to one single layer without overlays. The resulting 6 layers for the years 2007 to 2012 are then intersected with the district layer to calculate annual CAR coverage.

**Supplementary Figures**

**S1 Fig. Blacklisted districts and the blacklist criteria.**

The Venn diagram depicts the number of districts blacklisted and non-blacklisted from the first published list in 2008. Counts are based on PRODES official deforestation data. The blacklist was composed during the year 2008, therefore we consider for the first criterion the total deforested area until 2007. The first 36 districts with the highest deforested area fulfill criterion I. The first 36 districts with the highest deforested area between 2005 and 2007 fulfill the second criteria. All districts that at least show 3 years with increasing deforestation rates between 2003 and 2007 fulfill criterion III.

**S2 Fig. Forested districts of the Brazilian Legal Amazon.**

The map shows all districts of the Brazilian Legal Amazon (BLA), defined by INPE (771). In light grey are all districts with more than 10% forest cover in 2002 and complete information on all covariates used for the analysis (492). In dark grey are forested districts with incomplete data on the covariates (6).

**S4 Fig. Deforestation polygons to aggregate deforestation rates.**

Three detected deforestation polygons by satellite imagery are represented by the closed lines. The detection date of each polygon (end_date) represents the last date it could have been deforested. The first date an area could be deforested (start_date) is determined by the last satellite image that determined the polygon as forested. Annual deforestation rates are constructed by the sum of all polygons weighted by the share of the polygons’ timeframe within a given year.

**S4 Fig. Yearly total deforestation in the Brazilian Legal Amazon.**

The solid line shows yearly deforestation rates calculated by the INPE/PRODES project for the districts of the BLA (771). The dashed line shows deforestation rates calculated from INPEs shape files.

**S5 Fig. Parallel time trend assumption and potential biases conceptually.**

Panel a. shows the case of underestimating the impact due to selection bias where the real counterfactual of the blacklisted (had they not been treated) exhibits slower deforestation decreases than the used counterfactual, constructed from the control districts. Panel b. depicts the case of overestimating the impact where the real counterfactual of the treated districts would have had faster deforestation decreases than the used counterfactual (e.g., Ashenfelter’s dip).

**S1 Table. Data sources**

| **Variable** | **Year(s)** | **Source** |
| --- | --- | --- |
| Blacklist additions and removals | 2008-2012 | Decree 6.321/2007 and Provision 28/2008, Provision 102, 203/2009, Provision 66,67,68/2010 , Provision 138, 139, 175/2011, Provision 187,322,323,324/2012 |
| Deforestation and clouds | 2002-2012 | INPE-PRODES |
| Municipality list and borders | 2007 | IBGE |
| Protected areas | 2002-2012 | IBAMA |
| Indigenous areas | 2002-2012 | IBAMA |
| Settlement areas | 2002-2012 | INCRA |
| Mayors’ party affiliation | 2002-2012 | TSE |
| IPCA price deflator | 2002-2012 | IBGE |
| Soy prices | 2002-2012 | IBGE-PAM |
| Timber prices | 2002-2012 | IBGE-PEVS |
| GDP | 2002-2011 | IBGE |
| Number of farms | 2006 | IBGE Agricultural Census |
| Share of land owners | 2006 | IBGE Agricultural Census |
| Land value per ha | 2006 | IBGE Agricultural Census |
| Number of tractors | 2006 | IBGE Agricultural Census |
| Cattle stocking rate | 2006 | IBGE Agricultural Census |
| Population | 2007 | IBGE Demographic Census |
| Average distance to district center |  | Nelson |
| Field-based law enforcement inspections | 2001-2012 | IBAMA |
| Landholdings registered within the Cadastro Ambiental Rural (CAR) | 2002-2012 | Data base provided by the Amazon Environmental Research Institute (IPAM) in October 2013 |
| Rural credit | 2002-2012 | BCB |

**S2 Table.** Summary statistics on regression variables

|  | **N** | **Mean** | **SD** | **Min.** | **Max.** |
| --- | --- | --- | --- | --- | --- |
| **Time variant variables** | | | | | |
| Blacklisted | 5412 | 0.04 | 0.18 | 0.00 | 1.00 |
| Cloud error [share] | 5412 | 0.10 | 0.19 | 0.00 | 1.00 |
| Deforestation [sqkm] | 5412 | 28.30 | 68.82 | 0.00 | 1307.89 |
| GDP per capita [Reais] | 5412 | 9317.82 | 10439.87 | 1313.55 | 158972.89 |
| Soy price [Reais/kg] | 5412 | 0.13 | 0.28 | 0.00 | 1.99 |
| Timber price [Reais/cbm] | 5412 | 91.14 | 90.07 | 0.00 | 959.98 |
| Indigenous territory area cover [share] | 5412 | 0.08 | 0.17 | 0.00 | 1.00 |
| Multiple use protected area cover [share] | 5412 | 0.11 | 0.23 | 0.00 | 1.00 |
| Strictly protected area cover [share] | 5412 | 0.03 | 0.10 | 0.00 | 0.72 |
| Settlement area cover [share] | 5412 | 0.14 | 0.20 | 0.00 | 1.00 |
| Federal party affiliation | 5412 | 0.11 | 0.30 | 0.00 | 1.00 |
| **Time invariant variables** | | | | | |
| Initial total deforested area [sqkm] | 492 | 1087.84 | 1206.90 | 0.00 | 10253.15 |
| District area [sqkm] | 492 | 8667.78 | 15648.07 | 103.25 | 159522.59 |
| Farm area [sqkm] | 492 | 1641.25 | 2038.26 | 7.56 | 14576.02 |
| Population density [No./sqkm] | 492 | 21.30 | 97.32 | 0.09 | 1321.93 |
| Farms density [No./sqkm] | 492 | 0.59 | 0.93 | 0.00 | 12.30 |
| Share of small farms | 492 | 0.71 | 0.19 | 0.02 | 0.99 |
| No. of tractors per farm | 492 | 0.15 | 0.49 | 0.00 | 7.85 |
| Cattle rate [No./ha] | 492 | 1.50 | 2.13 | 0.00 | 31.94 |
| Share of land owners [%] | 492 | 73.34 | 23.17 | 4.49 | 100.00 |
| Land value [Reais/ha] | 492 | 1220.66 | 1007.49 | 80.00 | 7502.08 |

# Monetary figures are Million Brazilian Reais (BRL) deflated to 2012 prices, 1 BRL corresponded to USD 0.56 on average in 2012 ([**www.oanda.com**](http://www.oanda.com/)).

**S3 Table. Covariate balance before and after treatment**

| Covariate | Status | Mean blacklist | Mean non-blacklist | Difference in means | Normalized difference | Mean eQQ difference | % improvement mean difference |
| --- | --- | --- | --- | --- | --- | --- | --- |
| Total deforested area in 2007 | Unmatched | 3937.86 | 966.21 | 2971.65 | 1.21 | 2933.63 |  |
|  | Matched | 3937.86 | 2351.69 | 1586.17 | 0.65 | 1586.17 | 0.47 |
| Deforestation in 2005 | Unmatched | 141.02 | 14.68 | 126.35 | 1.00 | 125.10 |  |
|  | Matched | 141.02 | 57.41 | 83.62 | 0.66 | 83.62 | 0.34 |
| Deforestation in 2006 | Unmatched | 139.28 | 12.69 | 126.59 | 0.91 | 125.35 |  |
|  | Matched | 139.28 | 60.36 | 78.92 | 0.56 | 78.92 | 0.38 |
| Deforestation in 2007 | Unmatched | 125.55 | 12.28 | 113.27 | 0.75 | 111.43 |  |
|  | Matched | 125.55 | 62.11 | 63.44 | 0.42 | 63.44 | 0.44 |
| Deforestation increases | Unmatched | 2.38 | 1.70 | 0.68 | 1.07 | 0.74 |  |
|  | Matched | 2.38 | 2.44 | -0.06 | -0.09 | 0.06 | 1.09 |
| District area | Unmatched | 18106.38 | 7600.14 | 10506.24 | 0.42 | 9492.81 |  |
|  | Matched | 18106.38 | 12569.24 | 5537.14 | 0.22 | 5953.90 | 0.47 |
| Forest cover in 2007 | Unmatched | 0.58 | 0.46 | 0.12 | 0.63 | 0.16 |  |
|  | Matched | 0.58 | 0.54 | 0.04 | 0.20 | 0.07 | 0.69 |
| Population density in 2007 | Unmatched | 3.13 | 23.36 | -20.23 | -6.35 | 36.28 |  |
|  | Matched | 3.13 | 3.03 | 0.10 | 0.03 | 0.53 | 1.00 |
| Farms per sqkm in 2006 | Unmatched | 0.16 | 0.64 | -0.49 | -3.70 | 0.66 |  |
|  | Matched | 0.16 | 0.20 | -0.04 | -0.32 | 0.04 | 0.91 |
| Share of small farms in 2006 | Unmatched | 0.64 | 0.72 | -0.08 | -0.48 | 0.08 |  |
|  | Matched | 0.64 | 0.66 | -0.02 | -0.13 | 0.03 | 0.73 |
| Farm area cover in 2006 | Unmatched | 0.39 | 0.41 | -0.02 | -0.12 | 0.11 |  |
|  | Matched | 0.39 | 0.40 | -0.02 | -0.09 | 0.03 | 0.22 |
| No. of tractors per farm in 2006 | Unmatched | 0.31 | 0.14 | 0.17 | 0.37 | 0.27 |  |
|  | Matched | 0.31 | 0.19 | 0.12 | 0.25 | 0.12 | 0.32 |
| Cattle rate in 2006 | Unmatched | 1.18 | 1.54 | -0.36 | -0.86 | 0.87 |  |
|  | Matched | 1.18 | 1.26 | -0.08 | -0.19 | 0.10 | 0.78 |
| Share of land owners | Unmatched | 77.70 | 72.85 | 4.85 | 0.26 | 6.02 |  |
|  | Matched | 77.70 | 82.52 | -4.82 | -0.26 | 5.38 | 1.99 |
| Land value in 2005 | Unmatched | 1422.57 | 1197.82 | 224.74 | 0.30 | 379.43 |  |
|  | Matched | 1422.57 | 1429.67 | -7.10 | -0.01 | 184.18 | 1.03 |
| Av. distance to district center | Unmatched | 839.92 | 706.20 | 133.72 | 0.26 | 340.82 |  |
|  | Matched | 839.92 | 799.60 | 40.32 | 0.08 | 136.69 | 0.70 |
| Federal party affiliation in 2007 | Unmatched | 0.08 | 0.11 | -0.03 | -0.10 | 0.04 |  |
|  | Matched | 0.08 | 0.06 | 0.02 | 0.07 | 0.02 | 1.70 |
| GDP per capita in 2005 | Unmatched | 11381.30 | 7984.97 | 3396.33 | 0.43 | 4856.88 |  |
|  | Matched | 11381.30 | 9821.59 | 1559.71 | 0.20 | 1571.07 | 0.54 |
| GDP per capita in 2005 | Unmatched | 10131.02 | 8071.93 | 2059.09 | 0.40 | 3564.31 |  |
|  | Matched | 10131.02 | 9506.64 | 624.38 | 0.12 | 1448.08 | 0.70 |
| GDP per capita in 2005 | Unmatched | 12060.29 | 9062.95 | 2997.34 | 0.37 | 4161.11 |  |
|  | Matched | 12060.29 | 10353.12 | 1707.16 | 0.21 | 1859.55 | 0.43 |
| Indigenous territory cover | Unmatched | 0.14 | 0.07 | 0.06 | 0.37 | 0.08 |  |
|  | Matched | 0.14 | 0.11 | 0.03 | 0.15 | 0.03 | 0.60 |
| Multiple use protected area cover | Unmatched | 0.04 | 0.13 | -0.09 | -1.17 | 0.10 |  |
|  | Matched | 0.04 | 0.07 | -0.03 | -0.36 | 0.03 | 0.69 |
| Strictly protected area cover | Unmatched | 0.03 | 0.04 | -0.01 | -0.19 | 0.03 |  |
|  | Matched | 0.03 | 0.02 | 0.00 | 0.04 | 0.02 | 1.23 |
| Settlement area cover | Unmatched | 0.10 | 0.16 | -0.06 | -0.54 | 0.07 |  |
|  | Matched | 0.10 | 0.12 | -0.03 | -0.25 | 0.03 | 0.54 |

**References**
