# Supplementary material for: Naming and Shaming for Conservation: Evidence from the Brazilian Amazon
Source: PLoS One. 2015 Sep 23;10(9):e0136402. doi: 10.1371/journal.pone.0136402 (PMC4580616; doi:10.1371/journal.pone.0136402)
Supplement: S3 Text — (DOC) [file pone.0136402.s018.doc]

# Supplementary Information

**S3 Text. Parallel time trends of deforestation before treatment.**

**Testing for pre-treatment parallel trends in deforestation.** A key assumption in our analysis of blacklisting effects on deforestation is that forest loss would have followed the same trend in the blacklisted districts and the non-blacklisted districts in the absence of blacklisting. Since our data base covered the years from 2002-2007, we can test whether this has been the case before the blacklisting policy started in 2008. As per eq. 2 we rely on a first difference (FD) specification with log of deforestation as a dependent variable. Specifically, we thus assume equal growth trends in deforestation conditional on all covariates (see right panel of Fig. 3):

$E[\Delta\ln D_{it}|\Delta X_{it},B_{i}=1]=E[\Delta\ln D_{it}|\Delta X_{it},B_{i}=0]$ for $t=\{2002,...,2007\}$ Eq.2.1

We use two approaches to testing for pre-treatment equality of trends. The first test uses the full matched sample and the second (Chow’s test) compares trends among the blacklisted and non-blacklisted subsamples.

The full sample test relies on the following model using the notation from equations 1 and 2:

$\Delta\ln D_{it}=\beta\cdot B_{i}+\lambda_{1}t+\lambda_{2}t\cdot B_{i}+\Delta X_{it}'\gamma+Z_{i}'\delta+\kappa_{s}+\varepsilon_{it}$ for $t=\{2002,...,2007\}$ Eq.2.2

The deforestation growth trend for non-blacklisted districts is given by $\lambda_{1}$. The trend of the blacklisted districts is the linear combination of $\lambda_{1}+\lambda_{2}$. We use the Wald test for linear combinations to test the null-hypotheses that $\lambda_{1}=\lambda_{1}+\lambda_{2}$ or $0=\lambda_{2}$. The alternative hypothesis is $0\neq\lambda_{2}$, which we expect to reject if pre-treatment trends were equal.

For Chow’s test we first estimate the two models:

$\Delta\ln D_{it|B=1}=\lambda^{1}t+\Delta X_{it}'\gamma+Z_{i}'\delta+\kappa_{s}+\varepsilon_{it}$ Eq.2.3

$\Delta\ln D_{it|B=0}=\lambda^{0}t+\Delta X_{it}'\gamma+Z_{i}'\delta+\kappa_{s}+\varepsilon_{it}$ Eq.2.4

i.e., we run the same regression for blacklisted and matched non-blacklisted subsamples. To test if the time trends of both groups are equal we implement a Chow test to compare the two coefficients $\lambda^{1}$ and $\lambda^{0}$. In the case of equal pre-treatment deforestation growth trends we would fail to reject the Null-hypothesis that $\lambda^{1}=\lambda^{0}$.

**Results on the test of parallel time trends.** The estimation results of equations 2.2-2.4 are presented in columns 1-3 of S9 Table, respectively. Our first test produces an F-statistic of 2.63 and we thus cannot reject the null hypothesis of equal time trends with a p-value of 0.109. This means that the two coefficients a not significantly different from each other. Our second test shows the same result with a Chi-squared statistic of 1.30 and a p-value of 0.257. We also restricted the samples to years between 2003-2007, 2004-2007 and 2005-2007, to see if our assumption also holds for a fewer years before the blacklisting policy starts. Results are not reported as all tests produce equivalent results.

**S9 Table. Test for pre-treatment parallel time trends**

| Dependent | Δ ln Deforestation | | |
| --- | --- | --- | --- |
|  | (1) | (2) | (3) |
| Blacklisted_i_ | 127.197 |  |  |
|  | (78.503) |  |  |
| Year | -0.012 | -0.065*** | -0.016 |
|  | (0.033) | (0.025) | (0.026) |
| Blacklisted_i_ * Year | -0.063 |  |  |
|  | (0.039) |  |  |
| State effects | Yes | Yes | Yes |
| Time invariant controls | Yes | Yes | Yes |
| Time variant controls | Yes | Yes | Yes |
| Observations | 500 | 250 | 250 |
| Clusters | 76 | 50 | 26 |
| Adj. R-squared | 0.156 | 0.154 | 0.149 |

*Note:* The table reports first difference estimates with the dependent variable being the change in the log of yearly newly deforested area. Standard errors, clustered at district level, are reported in parentheses. Time invariant and variant controls include first differences of the variables reported in S2 Table. Observations are selected by a 1:1 closest neighbor matching using inverse-variance weights, with replacement. *** denote significance at the 1% level

**References**

1 Chow GC (1960) Tests of equality between sets of coefficients in two linear regressions. Econometrica: Journal of the Econometric Society: 591-605.
